# The COVID-19 pandemic and eating disorders in children, adolescents, and emerging adults: virtual care recommendations from the Canadian consensus panel during COVID-19 and beyond

**DOI:** 10.1186/s40337-021-00394-9

**Published:** 2021-04-16

**Authors:** Jennifer Couturier, Danielle Pellegrini, Catherine Miller, Neera Bhatnagar, Ahmed Boachie, Kerry Bourret, Melissa Brouwers, Jennifer S. Coelho, Gina Dimitropoulos, Sheri Findlay, Catherine Ford, Josie Geller, Seena Grewal, Joanne Gusella, Leanna Isserlin, Monique Jericho, Natasha Johnson, Debra K. Katzman, Melissa Kimber, Adele Lafrance, Anick Leclerc, Rachel Loewen, Techiya Loewen, Gail McVey, Mark Norris, David Pilon, Wendy Preskow, Wendy Spettigue, Cathleen Steinegger, Elizabeth Waite, Cheryl Webb

**Affiliations:** 1 McMaster University, Hamilton, ON Canada; 2 McMaster Children’s Hospital, 1200 Main St W, Hamilton, Ontario L8N 3Z5 Canada; 3 Canadian Mental Health Association – Waterloo Wellington, Waterloo, ON Canada; 4 University of Toronto, Toronto, ON Canada; 5 St. Joseph’s Care Group – Thunder Bay, Thunder Bay, ON Canada; 6 University of Ottawa, Ottawa, ON Canada; 7 The University of British Columbia, Vancouver, BC Canada; 8 University of Calgary, Calgary, AB Canada; 9 Ontario Ministry of Health and Long-Term Care, Toronto, ON Canada; 10 Dalhousie University, Halifax, NS Canada; 11 Laurentian University, Sudbury, ON Canada; 12 Patient advocate, Woodstock, ON Canada; 13 Parent advocate, Woodstock, ON Canada; 14 National Initiative for Eating Disorders, Toronto, ON Canada; 15 Parent advocate, Ottawa, ON Canada

**Keywords:** Guidelines, Eating disorders, COVID-19, Virtual care, Self-help, Anorexia nervosa, Bulimia nervosa, Binge eating disorder, Children, Adolescents, Emerging adults

## Abstract

**Objective:**

The COVID-19 pandemic has had detrimental effects on mental health. Literature on the impact on individuals with eating disorders is slowly emerging. While outpatient eating disorder services in Canada have attempted to transition to virtual care, guidelines related to optimal virtual care in this field are lacking. As such, the objective of our Canadian Consensus Panel was to develop clinical practice guidelines related to the provision of virtual care for children, adolescents, and emerging adults living with an eating disorder, as well as their caregivers, during the COVID-19 pandemic and beyond.

**Methods:**

Using scoping review methodology (with literature in databases from 2000 to 2020 and grey literature from 2010 to 2020), the Grading of Recommendations, Assessment, Development, and Evaluation system, the Appraisal of Guidelines, Research and Evaluation tool, and a panel of diverse stakeholders from across Canada, we developed high quality treatment guidelines that are focused on virtual interventions for children, adolescents, and emerging adults with eating disorders, and their caregivers.

**Results:**

Strong recommendations were supported specifically in favour of in-person medical evaluation when necessary for children, adolescents, and emerging adults, and that equity-seeking groups and marginalized youth should be provided equal access to treatment. For children and adolescents, weak recommendations were supported for telehealth family-based treatment (FBT) and online guided parental self-help FBT. For emerging adults, internet cognitive-behavioural therapy (CBT)-based guided self-help was strongly recommended. Weak recommendations for emerging adults included CBT-based group internet interventions as treatment adjuncts, internet-based relapse prevention Maudsley Model of Anorexia Nervosa Treatment for Adults (MANTRA) guided self-help, telehealth relapse prevention using MANTRA, and guided CBT-based smartphone apps as treatment adjuncts. For caregivers of children and adolescents, weak recommendations were supported for virtual parent meal support training, and moderated online caregiver forums and support groups. For caregivers of emerging adults, guided parental self-help CBT was strongly recommended, and unguided caregiver psychoeducation self-help was weakly recommended.

**Conclusions:**

Several gaps for future work were identified including the impact of sex, gender, race, and socioeconomic status on virtual care among children, adolescents, and emerging adults with eating disorders, as well as research on more intensive services, such as virtual day hospitals.

## Plain English summary

The objective of this project was to develop Canadian Practice Guidelines for the virtual treatment of children, adolescents, and emerging adults with eating disorders. We reviewed the literature for relevant studies, rated the quality of the scientific information within these studies, and then created recommendations for virtual care for children and adolescents (< 18 years), emerging adults (18–25 years), and their caregivers. We presented our key findings and proposed recommendations to a panel of clinicians, researchers, parents, and those with lived experience from across the country. Based on the discussions by the panel during our presentation, we revised the recommendations accordingly. This was followed by anonymous voting in an online survey, where panel members could vote and provide comments on the revised recommendations. Our final recommendations include strong recommendations for in-person medical evaluation, when necessary, for children, adolescents, and emerging adults, and that equity-seeking groups and marginalized youth should be provided equal access to treatment. For emerging adults, internet cognitive behavioural therapy (CBT)-based guided self-help was strongly recommended. Guided parental self-help CBT for caregivers of emerging adults was strongly recommended. For children and adolescents, weak recommendations were determined for telehealth family-based treatment (FBT), and online guided parental self-help FBT. For caregivers of children and adolescents, virtual parent meal support training, and moderated online caregiver forums and support groups were supported with weak recommendations. For emerging adults, weak recommendations were determined for CBT-based group internet interventions, internet-based relapse prevention Maudsley Model of Anorexia Nervosa Treatment for Adults (MANTRA) guided self-help, telehealth relapse prevention using MANTRA, guided CBT-based smartphone apps, and unguided caregiver psychoeducation self-help. The panel also identified several areas for future research, including the impact of sex, gender, race, and socioeconomic status on virtual care for eating disorders, as well as the need for further research on more intensive services such as virtual day hospitals.

## Introduction

The negative impact of COVID-19 and the associated social isolation on mental health has been well-described in terms of heightened anxiety and depression [1–3]. To cope with the stress of the pandemic, populations around the world are reporting changes in dietary patterns, such as increasing food intake or consuming more comfort food, and feeling anxious due to changed eating habits or activity levels [4, 5]. Survey results from a subset of the general population in Italy (*n* = 602), found that almost half of the respondents reported feeling anxious due to changed eating habits (e.g. consuming more comfort food in quarantine, and being inclined to increase food intake to feel better during COVID-19). During this time, females were also more prone to emotional eating than male counterparts [4]. In a survey in New Delhi, India, 79.5% of total respondents (*n* = 992) reported that their dietary pattern changed during the country’s lockdown [5]. This was especially true among people aged 35 to 50 years, who reported either increasing or decreasing their caloric intake (relative to before the pandemic) in response to stress related to social distancing laws and fears of acquiring COVID-19 [5].

Individuals with eating disorders (EDs) are also experiencing negative impacts related to COVID-19. A study in Spain found that after only 2 weeks of quarantine, nearly 40% of adults with EDs reported a worsening of ED symptomatology and almost 60% reported increased anxiety [6]. In the USA, Netherlands, and Australia, adults with Anorexia Nervosa (AN) reported increased restriction and fears about not being able to find foods consistent with their meal plan; those with Bulimia Nervosa (BN) and Binge Eating Disorder (BED) reported increases in binge eating and urges to binge; all respondents with EDs noted greater concerns about COVID-19 on their mental health than physical health, as well as concerns of ED relapse related to confinement [7, 8]. With closures of ED day hospitals occurring across Canada, Spain, Austria, USA, and the UK, the most severely ill ED patients were left without the intensive treatment they required [6].

There has been a lack of direction in terms of the best options for virtual ED care during the COVID-19 pandemic, and as a result, individuals, families, and clinicians are suffering the repercussions of health care systems that were ill-equipped for such a disruptive event [9]. As the ED population is particularly vulnerable and at significant risk of death should they not receive appropriate care [10], there is an urgent need to identify and prioritize the implementation of viable virtual care options.

Importantly, evidence indicates that telehealth is a promising alternative for the delivery of outpatient care [11]. The implementation of evidence-based treatments in mental health practice via video or teleconferencing, such as cognitive behavioural therapy (CBT) and family therapy, consistently demonstrates a comparable efficacy between online and face-to-face delivery, as well as similar therapeutic alliance and satisfaction between these delivery modes [see reviews for CBT [12–15] and family therapy [16, 17]]. The telehealth format, however, can present significant challenges for ED care with respect to developing rapport with individuals with EDs, as well as engaging in an appropriate assessment of medical stability. Reports have been made by adults with AN, BN, and BED in the USA and Netherlands about the quality of ED treatment during the pandemic being “somewhat” or “much” worse than usual care [7]. While some people with EDs may be willing to engage in virtual therapy, a preference for in-person care remains high [18]. Individuals with EDs often experience ambivalence regarding video calls, reporting a heightened self-criticism and awareness of bodily appearance, which respondents indicate creates a negative experience during virtual visits [6]. Furthermore, individuals with EDs and caregivers have voiced concern over the lack of face-to-face accountability with virtual weigh-ins, as well as concerns that social isolation may result in long periods of time to engage in excessive exercise [6, 19].

Technologies such as mobile applications (‘apps’), self-help resources, and web-based information can be important sources of support during COVID-19 [18, 19]. However, social media posts and advertisements continue to bombard users with messages about “inevitable” weight gain related to staying at home, and have been argued to promote shape and weight concerns as well as restrictive and compensatory behaviours among those with, or at-risk for, EDs [20–23]. News media reports of food scarcity can cause individuals with EDs to restrict further due to a sense of altruism, or households maintaining a week-long supply of groceries can be a trigger for binge eating episodes [24].

Our team recently published *Canadian Practice Guidelines for the treatment of children and adolescents with EDs* [25]; however, many of the recommended interventions are focused on in-person individual and family treatment or group therapy, which currently cannot be delivered. Common challenges of delivering evidence-based treatment for EDs, as well as possible solutions and practical considerations have been recently published [26], taking into consideration the COVID-19 pandemic, yet formal guidelines are lacking. The present project represents an addendum to our guidelines and focuses on the generation of recommendations related to the provision of effective virtual care and online support for children, adolescents, and emerging adults who are living with an ED, and their caregivers. We expect these guidelines to have relevance for clinicians, administrators, and policymakers wishing to provide the best possible ED care during the COVID-19 pandemic and beyond.

### Objectives

Our aim was to synthesize the best available evidence on: a) the impact of COVID-19 on children, adolescents, and emerging adults with EDs, and their caregivers, and, b) virtual treatments and other supports and technologies for this population. The research questions listed below were created and discussed by our research team and guideline development panel, consisting of clinicians (e.g. health care professionals), researchers, knowledge users, and patient/parent advocates from across Canada.

### Research questions

In children, adolescents and emerging adults with EDs and their caregivers: 1) What is the impact of COVID-19? 2) What evidence exists for treatments that can be delivered virtually? 3) What evidence exists for self-help for affected individuals and caregivers? 4) What evidence exists for other e-technology (email therapy, text messaging therapy, smartphone apps)? 5) What evidence exists for virtual day hospital, virtual group therapy, and virtual meal support? 6) When should individuals with EDs be seen in-person for evaluation? And how can medical monitoring be done at home? 7) How do sex and gender impact virtual care? and, 8) What are the gaps in the research evidence?

## Methods

### Overview

Since it was expected that the literature would be limited but diverse, we used scoping review methodology [27–30] to ensure we collated all evidence on the impact of COVID-19 on the ED population, virtual ED treatment in the COVID-19 context, and research focusing on other novel technologies, online self-help support, and individual/caregiver experiences with online approaches. This was followed by a grading of the evidence using the *Grading of Recommendations, Assessment, Development, and Evaluation (GRADE)* system [31–33]*.* These evidence profiles were then presented to a panel of stakeholders from across Canada, followed by a voting system and arrival at consensus on the recommendations. The *Appraisal of Guidelines, Research and Evaluation (AGREE II)* tool was used to inform guidelines development and reporting [34]. In addition to recommendations based on existing research evidence, recommendations were also made representing the consensus of the panel with respect to good clinical practice. Preferences, implementability factors, and previous experiences of the expert panel were considered when making each recommendation, in combination with research evidence, or in place of research evidence where none existed.

Virtual care is a broad term which encompasses all the methods in which healthcare providers remotely interact with their patients [35]. Telemedicine is one of these methods, as are a variety of video and audio methods that we included in our literature search. The World Health Organization (WHO) defines telemedicine as: “the delivery of health care services at a distance, by health care professionals using information and communication technologies related to diagnosis, treatment, evaluation, research, or education, all in the interest of advancing health care” [36]. Generally, the terms ‘telemedicine’ and ‘telehealth’ are synonymous and can be used interchangeably [36]. Virtual care can be synchronous, involving the use of audiovisual technology in real-time for patient and health care professional communication, or asynchronous, consisting of health information being collected at one location and virtually transferred to another for review by a health professional [37]. The panel mutually agreed on including various synchronous and asynchronous virtual modalities including telehealth/telemedicine, telephone support services, text messaging, smartphone apps, email, e-mental health platforms (e.g. mental health services delivered via the internet or other digital technologies), self-help, and moderated online forums. The panel decided to exclude social media as there was consensus that social media were not an overt method of treatment/support (and likely have a mix of benefit and harm). Additionally, given the volume of literature that exists related to social media and EDs, and the short timeline of our project, it was decided it was not feasible to include this literature.

### Synthesis methods

#### Eligibility criteria

Our inclusion criteria were: a) all literature, including quantitative, qualitative, and mixed methods papers on the impact of COVID-19, as well as virtual and online treatments/supports for children/adolescents (< 18 years) and emerging adults (18–25 years) with EDs and/or their caregivers; and b) articles written in any language. The inclusion of emerging adults differs from our previous *Canadian Practice Guidelines* [25], which only included children and adolescents (< 18 years). Prior to the present literature search, the panel unanimously agreed to include a target population of up to and including 25 years of age, where those aged 18 to 25 years were defined as emerging adults. This was decided so that studies on the transition of services from pediatric to adult services, which may involve virtual components, could be reviewed. The panel also anticipated that there would be little research on virtual treatments for those under 18 years of age, and that most research would focus on the 18 to 25 age range. During the screening process, the citation reviewers agreed to include studies whose participants had a mean age of up to and including 25 years. This was not ideal, as means are heavily influenced by sample size and range of values***.*** Many of the studies also did not include an upper age limit, or if they did it was greater than 25 years. Despite these concerns, the citation reviewers agreed that including studies with a mean age of up to and including 25 years ensured that participants that did meet our target age group would be accounted for in our findings and ultimately, in the recommendations. Our exclusion criteria were: a) studies primarily involving adults (> 25 years); and b) studies that did not include EDs or disordered eating behaviours.

### Identifying potentially eligible studies

#### Databases

A literature search was completed using the following databases: Medline, PsycINFO, EMBASE, Cochrane Database of Systematic Reviews, Cochrane Central Register of Controlled Trials (CENTRAL), and CINAHL. The references of relevant articles obtained were also reviewed.

#### Literature search strategy

Our library scientist (NB) designed and executed comprehensive searches in the databases listed above to obtain evidence for each of the research questions dating back the last 20 years (2000 to 2020). This time frame was chosen based on the fact that there was likely little or no virtual care prior to 20 years ago and based on feasibility. The searches included a combination of keyword and subject heading for each concept. The sample search strategy included, but was not limited to, various combinations of the following terms as appropriate for the questions being addressed: Anorexia Nervosa OR Bulimia Nervosa OR Eating Disorder Not Otherwise Specified OR eating disorder OR Other Specified Feeding and Eating Disorder OR Avoidant/Restrictive Food Intake Disorder OR Binge Eating Disorder OR Atypical Anorexia Nervosa; AND virtual care OR self-help OR telemedicine OR telehealth OR videoconferencing OR COVID-19 OR coronavirus OR pandemic.

#### Other strategies

Grey literature was also reviewed, including conference proceedings from the International Conference on Eating Disorders between 2010 to 2020. Databases of ongoing research were searched including CENTRAL and clinicaltrials.gov. We also hand searched the International Journal of Eating Disorders from the last 10 years (2010 to 2020) for relevant articles.

### Applying eligibility criteria and extracting data

Two members of the research team independently evaluated the results generated by our searches and came to consensus on which studies met eligibility criteria. We used Endnote and DistillerSR software to organize our studies. Duplicate records were removed. DistillerSR was used for article screening and data extraction. Titles and abstracts were used to exclude obviously irrelevant reports by the two reviewers. Potentially relevant articles were reviewed in full text by two reviewers who had to agree on inclusion. Articles in other languages were translated into English using Google Translate (*n* = 6). References of included reviews and book chapters were examined to find other potentially relevant studies. There were no disputes. Data extraction included the indexing of the type of paper, type of control group (if any), methodology, type of virtual intervention, ED diagnosis, age range, sample size, description of intervention, outcomes, results, and whether or not the paper described sex and/or gender as impacting virtual care. Sex was defined as sex assigned at birth, categorized into male or female. Gender was defined as the individual’s self-identified gender role/identity categorized as girl, boy, cis, trans, or other gender identities.

### Appraising studies and guideline-related frameworks

The *GRADE* system [31–33] explicitly describes how to rate the quality of each study, as well as how to synthesize the evidence and grade the strength of a recommendation. Using this system, we used GRADEpro software to develop an evidence profile for intervention studies where treatment outcomes could be summarized in this fashion. With GRADEpro, we synthesized and classified the overall quality of evidence for each intervention based on the quality of all of the studies, taking into account risk of bias, inconsistency, indirectness, imprecision, publication bias, dose-response, and effect size [32]. Although we examined each outcome independently, when the rating of the evidence was the same, we collapsed the treatment outcomes in the GRADEpro tables for the sake of efficiency. We also used the *AGREE II* tool as well as the *Guideline Implementability for Decision Excellence Model (GUIDE-M)* to inform guideline development and this report [38, 39].

### The guideline team

The Guideline Team is comprised of a core research team and a larger guideline development panel (GDP). This team is skilled in guideline development having just published the first *Canadian Practice Guidelines for the treatment of children and adolescents with EDs* [25]. The research team presented the research questions to the GDP, reviewed evidence summaries, formulated practice recommendations, drafted the guideline, and limited biases that could impeach upon the guideline development process [40–42]. The chair of the GDP (MB) is a methodological expert in guideline development, with content expertise outside the field of eating disorders. She led the consensus discussions of the GDP and oversaw conflict-of-interest disclosures and management. Our multi-disciplinary GDP of 27 diverse stakeholders from across Canada included experts in the field of EDs, multi-disciplinary front-line clinicians/knowledge users, those with lived experience (parents and individuals), hospital administrators, and policymakers (all authors on this guideline).

### Procedures

An initial videoconference (via Zoom) was held on May 29, 2020 with the Guideline Team. The videoconference was recorded to provide an opportunity to review at a later date if necessary. The aims of the videoconference were to confirm the research questions, review the guideline development process and roles and responsibilities of the GDP, and identify potential conflicts of interest. During the meeting, the research questions were refined, the clinical population and outcomes were discussed, and the target audience was reviewed.

Once the literature searches were completed and the evidence profiles generated, a second videoconference (via Zoom) was held on September 25, 2020, which was also recorded so it could be reviewed if required. Two members of the core research team (JC and DP) presented evidence profiles for discussion with the GDP. This was followed by a facilitated discussion of the evidence profiles and draft recommendations generated by the core team. For each question, the panel reviewed the evidence, and discussed: (i) whether the interpretation of the evidence put forward by the core team aligned with that of the GDP; (ii) strengths and limitations of the evidence base; and (iii) considerations of the generalizability of the studies, precision of the estimates, and whether the evidence aligned with values and preferences of Canadian individuals with EDs and clinicians. Alternative interpretations and suggestions for further research were discussed. Minor or dissenting opinions were noted. Issues regarding implementability of the recommendations were considered, and suggestions for dissemination of the guidelines were elicited. In terms of formulating the recommendations, the panel generally defined a recommendation as ‘weak’ if it was supported by low certainty evidence (e.g. case report, case study, open trial data); a recommendation was defined as ‘strong’ if it was supported by high certainty evidence (e.g. randomized controlled trial data), while also taking into consideration all of the contextual factors mentioned above. Additionally, the recommendation could be formulated as ‘for’ or ‘against’ the intervention.

Following the virtual meeting, GDP members were provided with the draft guidelines for review. Group consensus on recommendations and strength of recommendations was obtained by using a modified Delphi method [43], with voting by all team members (except JC, DP, MB and NB) using an anonymous web-based survey platform, Lime Survey (www.limesurvey.com). For a recommendation to be approved, at least 70% of the GDP were required to identify their agreement with the recommendation [33]. Consensus was achieved in the first round of voting.

## Results

### The impact of COVID-19 on eating disorders

**Fig. 1:**
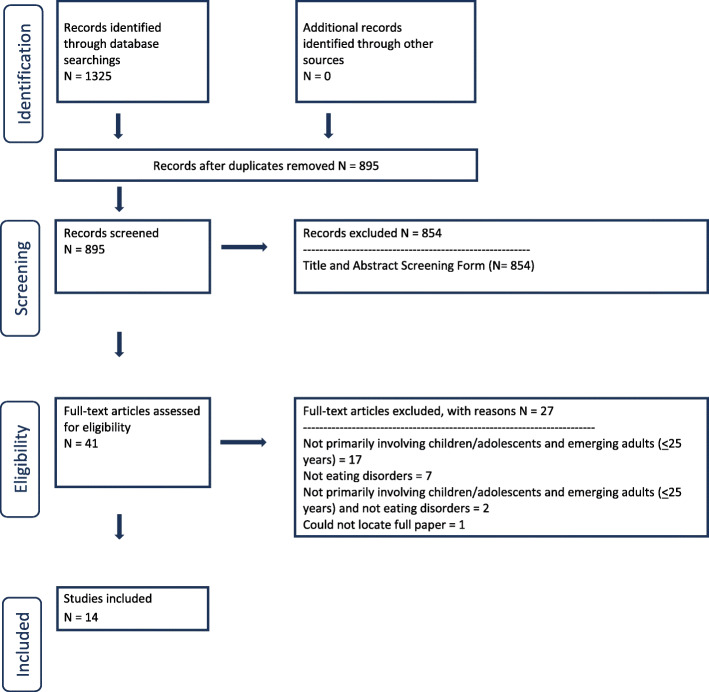
PRISMA flow diagram for the impact of COVID-19 on children/adolescents and emerging adults with EDs, as well as their families and clinicians

One thousand, three hundred and twenty-five abstracts were identified for review within the impact of COVID-19 on EDs section of our guideline (see PRISMA flow diagram, Fig. 1). After duplicates were removed, abstracts screened, and full-text articles reviewed, 14 papers were included within this section. No additional abstracts were identified through review of reference lists. See Appendix A for a summary of the included studies regarding the impact of COVID-19 on EDs.

#### Treatment modifications and/or ED symptoms experienced

##### Children/adolescents

Some evidence described the impact of COVID-19 on children and adolescents, and/or their caregivers and clinicians. Four descriptive studies revealed a rapid scale-up of telehealth for adolescents in ED programs as a result of the pandemic [26, 44–46]. According to clinicians, telehealth created some challenges, including difficulties in ensuring accurate communication when monitoring remotely, privacy concerns, difficulties building rapport with individuals with EDs, individuals with EDs not being physically present for weigh-ins, issues ensuring family involvement in Family-Based Treatment (FBT) sessions, inability to oversee mealtime, and individuals with EDs and/or parents disconnecting or leaving video sessions unexpectedly [26]. COVID-19-related modifications to treatment led to an increased workload experienced by clinicians and an increased caregiver burden (e.g. parents having to weigh their child at home) [45, 46]. Despite these challenges, in one descriptive study that featured a case report, the individual with an ED (*n* = 1) achieved calorie goals, reduced purging episodes and emotional outbursts, engaged in telehealth care, and avoided re-hospitalization [44].

##### Emerging adults

Like children and adolescents, descriptive study evidence revealed an increase from 0 to 97% in 1 month for telemedicine visits for emerging adults with EDs in an Adolescent and Young Adult Medicine Clinic, and that this population in particular benefitted from telemedicine [47]. Challenges included privacy issues and inability to assess anthropometric data [47]. If weights and vital signs were unable to be collected at home by caregivers, partnerships with local primary care providers were formed to collect data [47, 48]. One qualitative study outlined themes of the impact of COVID-19 treatment modifications for individuals with EDs and caregivers [49]. For individuals the themes included: reduced access to ED services, varying levels of acceptability for remote therapy, reduced motivation for recovery, heightened psychological distress and ED symptoms, and increased attempts at self-management in recovery. For caregivers the themes included: fears of premature discharge from treatment, increased caregiver burden, managing new challenges around patient well-being (e.g. spotting signs of relapse), and new opportunities (e.g. gratitude for increased time at home).

#### Impact on clinicians treating individuals with EDs during COVID-19

##### Clinicians caring for individuals with EDs of any age

Six articles [50–55], provided suggestions for clinicians for ED care and research during the COVID-19 pandemic, and one article [18] described the impact on clinicians working with individuals with EDs during this time.

Two descriptive studies provided recommendations to combat the challenges associated with remote delivery of ED care [50, 51]. These included stressing telehealth sessions were ‘business as usual’ to individuals with EDs who might view telehealth as ‘second best’, using reliable video-call platforms instead of audio calls to deliver virtual treatment, acknowledging the challenges associated with COVID-19 to those with EDs, and having COVID-19-specific ED psychoeducation for CBT or enhanced CBT (CBT-E) sessions. Other descriptive research suggested clinicians consider a different approach to ED treatment during the COVID-19 pandemic, such as implementing a person-centered and harm-reduction approach [52], or specifically for individuals with Avoidant/Restrictive Food Intake Disorder (ARFID), fostering communities of kindness towards ARFID that would involve carefully listening to individuals, family members, and partners of those affected by the disorder and COVID-19 [53]. For research, one editorial [54] and one cross-sectional study [55] indicated a need to fast-track ED research and publications related to COVID-19. Additionally, it was recommended that research disruptions be handled by employing technology, reprioritizing study goals (e.g. changing research directions), and encouraging collaboration between sites [54, 55].

In terms of clinician impact, one editorial revealed clinicians’ concern over e-technology adding to their workload, where there may be a new expectation for them to handle patient-related issues during off-work hours given accessiblity to virtual patient data and inquiries [18].

### Virtual care and eating disorders

**Fig. 2:**
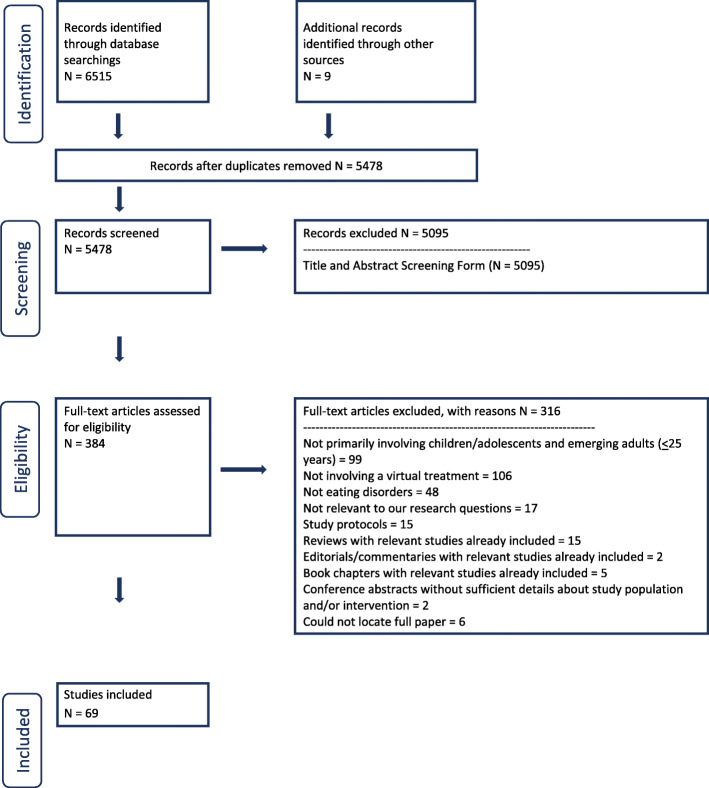
PRISMA flow diagram for the telehealth virtual care and eating disorders for children/adolescents and emerging adults

Six thousand, five hundred and fifteen abstracts were identified for review within the virtual care and EDs section of our guideline (see PRISMA flow diagram, Fig. 2). Nine additional abstracts were identified through review of reference lists. After duplicates were removed, abstracts screened, and full-text articles reviewed, 69 studies were included within this section of our guideline. See Appendix B for a summary of the included studies regarding virtual care and EDs. Two distinct groups of treatments were found: established treatments used routinely face-to-face being delivered virtually (e.g. via video or teleconferencing), and interventions designed specifically for remote use delivered in a variety of ways. In terms of evidence related to treatments traditionally delivered face-to-face, we did not report on all of this research, but we did consider the existence of this evidence base when creating our recommendations.

### Telehealth using synchronous videoconferencing and/or teleconferencing

#### Family-based treatment (FBT)

##### Children/adolescents

Two studies were found using telehealth FBT, which followed an FBT manual but involved therapists virtually assisting parents to support nutritional recovery of their child via a telehealth platform or telephone, in children and adolescents with AN or atypical AN [56, 57]. In a feasibility study (*n* = 10), examining the preliminary effect of telehealth FBT on weight gain [56], significant weight gain was achieved from baseline to end of treatment and at follow-up (with moderate to large effect sizes). Significant weight gain was also achieved from baseline to end of treatment in a case report (*n* = 1) for telehealth FBT [57] (Table 1).

**Table 1:** Telehealth FBT for children and adolescents

Certainty assessment							Impact	Certainty	Importance
№ of studies	Study design	Risk of bias	Inconsistency	Indirectness	Imprecision	Other considerations			
**Outcomes: Weight gain**									
2	observational studies	very serious^a,b^	not serious	not serious	not serious	strong association^c^	1 feasibility open trial [56] and 1 case report [57] with children with AN (total *n* = 11). Significant weight gain was achieved in both studies, from baseline to end of treatment and/or at follow-up.	⨁⨁⨁◌ MODERATE	CRITICAL

^a^No control condition

^b^No randomization

^c^The open trial [56] had moderate to large effect sizes for participant weight gain from baseline to end of treatment and from baseline to 6-month follow-up

Bibliography:

Observational studies: Open trial – Anderson 2017 [56]; Case report – Goldfield 2003 [57]

#### Relapse prevention (MANTRA)

##### Emerging adults

In one open trial using a post hospitalization sample of individuals with AN, trial completers (*n* = 12) in the Maudsley Model of AN Treatment for Adults (MANTRA) intervention (consisting of 10 sessions of a relapse prevention programme for AN over 4 months) delivered via telehealth demonstrated increased Body Mass Index (BMI) and lower eating, shape and weight concerns (Eating Disorder Examination – Questionnaire [EDE-Q] scores), compared to baseline [58] (Table 2). This was a pilot study, examining the subjective need for a relapse prevention intervention, the feasibility and acceptance of the intervention and delivery via videoconference, as well as, exploratory measures of change in weight, relapse rates, ED pathology, and transition to outpatient care.

**Table 2:** Telehealth relapse prevention using MANTRA for emerging adults

Certainty assessment							Impact	Certainty	Importance
№ of studies	Study design	Risk of bias	Inconsistency	Indirectness	Imprecision	Other considerations			
**Outcomes: BMI; EDE-Q**									
1	open trial	very serious^a,b^	not serious	not serious	not serious	none	1 pilot open trial with emerging adults with AN (*n* = 16) [58]. BMI increased after the 4-month intervention.	⨁⨁◌◌ LOW	CRITICAL
							Programme completers (*n* = 12) had significantly lower eating, shape, and weight concerns (EDE-Q scores) at the end of the intervention vs. baseline [58].		

^a^No control condition

^b^No randomization

Bibliography:

Open trial – Giel 2015 [58]

#### Cognitive behavioural therapy (CBT)

##### Children/adolescents

Case report evidence was found for telehealth cognitive and behavioural treatment for ARFID and Other Specified Feeding and Eating Disorder (OSFED); however, both studies had small sample sizes [59, 60]. For instance, in one case report, teleconsultations between clinicians and caregivers related to feeding interventions for ARFID resulted in an increase in the frequency of bites of nonpreferred foods consumed, though results were for one individual only (8-year-old male) [59]. Likewise, another case report involving CBT telepsychologist visits (using videoconferencing platforms) for Eating Disorder Not Otherwise Specified (EDNOS) resulted in an increase in food intake, improvements in growth, and reduced depression scores; however, again the case report only studied one individual (14-year-old female) [60].

### Self-help and guided self-help

### Guided self-help

#### Internet CBT-based guided self-help

##### Emerging adults

Of all self-help treatments examined, internet CBT-based guided self-help had the most evidence to support its use in emerging adults with AN, BN, BED, and EDNOS. Three RCTs [61–63] and their subsequent studies [64–67] demonstrated reduced ED psychopathology, improvements in ED symptoms (e.g. cessation from binge eating and purging), and/or significant weight gain among various internet CBT-based guided self-help interventions (‘Featback’ [61], ‘Overcoming Bulimia Online’ [62], and ‘VIA-Virtual Intervention for AN’ [63]) compared to controls. Of these, one RCT that compared intensities of virtual therapist support (low = one time per week versus high = three times per week) in addition to the internet-based program (‘Featback’) revealed no added value of therapist support in symptom reduction, but the added support contributed to greater program satisfaction [61] (Table 3).

**Table 3:** Internet CBT-based guided self-help for emerging adults

Certainty assessment							Impact	Certainty	Importance
№ of studies	Study design	Risk of bias	Inconsistency	Indirectness	Imprecision	Other considerations			
**Outcomes: ED psychopathology (SEED, EDE-Q)**									
2	randomized trials	not serious	not serious	not serious	not serious	none	1 RCT [61] and a subsequent study [65] with emerging adults with AN, BN, BED, and EDNOS (total *n* = 87 Featback; *n* = 88 Featback + low-intensity therapist support; *n* = 89 Featback + high-intensity therapist support; *n* = 90 waitlist control). Baseline levels of ED psychopathology were found to moderate intervention response. The 3 Featback conditions were superior to waiting list control in reducing bulimic psychopathology (SEED and EDE-Q scores). No added value of therapist support was found in symptom reduction but did improve intervention satisfaction; no significant differences between Featback conditions, and no effects were found regarding anorectic psychopathology.	⨁⨁⨁⨁ HIGH	CRITICAL
**Outcomes: costs (related to intervention, health care utilization, medication; assessed using Health and Labor Questionnaire)**									
1	randomized trials	not serious	not serious	not serious	not serious	none	1 subsequent study [64] to the Featback RCT [61]: no significant differences between the study conditions were found regarding societal costs. Mean costs per participant were lowest in the Featback condition with low-intensity therapist support, followed by Featback with high-intensity therapist support, Featback without therapist support, and waiting list. Featback seems to be cost-effective vs. waitlist.	⨁⨁⨁⨁ HIGH	CRITICAL
**Outcomes: BMI (Weight gain)**									
2	randomized trials	not serious	not serious	not serious	not serious	none	1 RCT with individuals with AN (*n* = 128 VIA intervention; *n* = 130 control) for relapse prevention [63]. Intervention completers gained significantly more weight than treatment as usual controls. At 9-month follow-up of this RCT [67] (at 9-month follow-up, *n* = 92 VIA intervention; *n* = 120 control), very good results for BMI were seen for full completers of the intervention. Predictors for favourable course (concerning BMI) were adherence to intervention, more spontaneity, and better self-esteem.	⨁⨁⨁⨁ HIGH	CRITICAL
**Outcomes: dropout rate**									
1	randomized trials	not serious	not serious	not serious	not serious	none	A subsequent study [66] to the VIA RCT [63] reported VIA was well-received and highly feasible with a moderate dropout rate (15.5%).	⨁⨁⨁⨁ HIGH	CRITICAL
**Outcomes: ED symptoms (frequency of binge eating, vomiting, etc.)**									
5	observational studies	very serious^a,b^	serious^c^	not serious	not serious	Strong association^d^	2 open trials (total *n* = 228), 1 controlled study (*n* = 31 intervention; *n* = 31 waitlist control), 1 case series (*n* = 38), 1 case report (*n* = 1) all with those with BN and/or EDNOS. Both open trials had significant improvements in ED symptoms at follow-up [68, 69]. In the controlled study, binge eating and vomiting abstinence rates differed significantly between the internet and control groups at post-treatment, favouring the internet group [70]. The case series saw significant decreases in vomiting and weight phobia, but when bingeing and vomiting decreased, exercise increased [71]. The case report did not see an improvement in ED symptoms during the intervention, although it involved ProYouth, which is used for ED prevention and early intervention [72].	⨁⨁◌◌ LOW	CRITICAL
1	randomized trials	not serious	not serious	not serious	not serious	none	1 RCT with BN and EDNOS participants (*n* = 38 Overcoming Bulimia Online intervention; *n* = 38 waitlist/delayed treatment control) [62]. The intervention group had higher rates of cessation from binge eating and purging vs. delayed treatment condition, who experienced little change in cessation rates at follow-up. Intervention group gains were maintained or continued to improve at follow-up.	⨁⨁⨁⨁ HIGH	CRITICAL

^a^No control condition

^b^No randomization

^c^Some discrepancies between study findings

^d^Large effect sizes for changes in ED symptoms scale [70] and weight phobia [71] and from baseline to post-treatment.

Bibliography:

RCTs – Aardoom 2016 [61], Aardoom 2017 [65], Aardoom 2016 [64], Fichter 2012 [63], Fichter 2013 [67], Fichter 2011 [66], Sanchez-Ortiz 2011 [62]

Observational studies: Open trials – Pretorius 2009 [68], Carrard 2011 [69]; Controlled study – Fernandez-Aranda 2009 [70]; Case series – Nevonen 2006 [71]; Case report – Kindermann 2016 [72]

Non-randomized studies saw mixed results for individuals with BN and/or EDNOS and internet CBT-based guided self-help programs. Two open trials (total *n* = 228) observed significant improvements in ED symptoms (e.g. decreased objective binge eating and vomiting) and general psychopathology following online CBT-based intervention programs [68, 69]. One controlled study revealed significant decreases at follow-up in psychopathological levels, binge eating, and vomiting, favouring the intervention group (*n* = 31) over waitlist controls (*n* = 31) [70]. In contrast, one case series found that for participants (*n* = 38) who followed an internet CBT-based guided self-help program, there was a notable change in their methods of compensation rather than an actual improvement in behaviours (e.g. participants decreased vomiting frequency but increased excessive exercise episodes) [71]. Similarly, one case report with one individual observed no improvements in BN symptoms following a different internet CBT-based intervention (‘ProYouth’) [72] (Table 3).

In terms of qualitative findings, two qualitative studies reported that an internet CBT-based guided self-help intervention (‘Overcoming Bulimia Online’) was well-received among BN and EDNOS participants (total *n* = 20); these favourable perceptions of the program were attributed to the programs’ accessibility, flexibility, support, and content [73, 74]. Notably, some participants used the program as a ‘stepping-stone’ to further treatment [73].

#### CBT-based Bibliotherapy

##### Emerging adults

One RCT [75] and its subsequent studies [76, 77] compared internet-guided self-help (INT-GSH) and bibliotherapy-guided self-help (BIB-GSH) among emerging adults with BN and/or EDNOS (*n* = 70 INT-GSH, *n* = 56 BIB-GSH) and reported significant improvements in ED symptoms in both groups. There was no significant difference regarding outcome between delivery modes of the treatment, and authors suggested that both may be equally effective treatment options for this population (Table 4).

**Table 4:** CBT-based bibliotherapy for emerging adults

Certainty assessment							Impact	Certainty	Importance
№ of studies	Study design	Risk of bias	Inconsistency	Indirectness	Imprecision	Other considerations			
**Outcomes: ED symptoms (frequency of binge eating, compensatory measures, etc.); ED psychopathology (EDI-2 scores)**									
3	randomised trials	not serious	not serious	not serious	not serious	none	1 RCT [75] and its subsequent studies [76, 77] with emerging adults with BN and EDNOS (total *n* = 70 INT-GSH; total *n* = 56 BIB-GSH). ED symptoms (objective binge eating and compensatory behaviour) improved significantly in both groups. Greatest improvements in ED symptoms were after 4 months; by month 18, 14.6% (7/48) of INT-GSH participants and 25% (7/28) of BIB-GSH participants were abstinent from binge eating and compensatory measures. There were no significant differences in outcomes found between the 2 groups. In both groups, lower frequency of binge eating at baseline predicted good outcomes at long-term follow-up (18 months).	⨁⨁⨁⨁ HIGH	CRITICAL
							There were no group differences (between INT-GSH and BIB-GSH) in EDI-2 subscales [75].		

Bibliography:

RCTs – Wagner 2013 [75], Wagner 2013 [76], Wagner 2015 [77]

#### Manual CBT-based guided self-help

##### Emerging adults

One observational controlled study with individuals with AN studied a manualized CBT-based guided self-help intervention (*n* = 51) with exercises to practice coping skills and improve body image in addition to weekly telephone contact with a clinical psychologist, versus a waitlist control (*n* = 51) [78]. Both groups thereafter received inpatient treatment. Duration of inpatient treatment was significantly shorter (by an average of 5.2 days) among intervention participants compared to controls. Body image, slimness ideal, general psychopathology and atypical binges improved significantly during the guided self-help intervention; however, while the intervention group showed more weight gain, changes in BMI did not differ significantly between the groups.

#### Internet-based relapse prevention MANTRA guided self-help

##### Emerging adults

One RCT of individuals with AN studied internet-based MANTRA (iMANTRA) guided self-help (MANTRA workbook and email support from a therapist) in addition to treatment as usual (*n* = 24) versus a treatment as usual only control group (treatment from a local community mental health team or child/adolescent mental health team; *n* = 17) [79]. This feasibility study examined the use of email guided self-care treatment added to treatment as usual. This was a relapse prevention study post hospital or day treatment, and a key aim of iMANTRA was to facilitate patient engagement in ongoing outpatient treatment, so the generalizability of this study to other populations is limited. At 6 months, there was little difference between the groups in terms of outcomes, but at 12 months, the iMANTRA intervention group had a higher BMI and lower Depression, Anxiety, and Stress Scale (DASS-21) scores than treatment as usual controls; the iMANTRA group also had fewer readmission rates than the treatment as usual control group. Despite the differences between groups at 12 months, confidence intervals were wide and overlapped with zero, which decreases the certainty of the findings (Table 5).

**Table 5:** Internet-based MANTRA guided self-help for emerging adults

Certainty assessment							Impact	Certainty	Importance
№ of studies	Study design	Risk of bias	Inconsistency	Indirectness	Imprecision	Other considerations			
**Outcomes: BMI; Depression Anxiety and Stress Scale (DASS-21)**									
1	randomised trial	not serious	not serious	not serious	not serious	none	1 feasibility RCT with AN participants (*n* = 24 internet-MANTRA +treatment as usual; *n* = 17 treatment as usual) [79]. At 6 months: little difference between iMANTRA group (internet-based self-help workbook + email support by therapist for AN relapse prevention) and treatment as usual group. At 12 months: iMANTRA group had a higher BMI than treatment as usual group, but confidence intervals were wide and overlapped with 0.	⨁⨁⨁⨁ HIGH	CRITICAL
							At 12 months, iMANTRA group had lower DASS-21 scores compared to treatment as usual group [79].		

Bibliography:

RCT – Schmidt 2017 [79]

### Unguided self-help

#### Internet-delivered self-compassionate letter writing unguided self-help

##### Emerging adults

One RCT with individuals with AN and atypical AN in an internet-delivered self-compassionate letter-writing intervention (adapted from compassion-focused therapy) for non-treatment seeking individuals (*n* = 20) resulted in greater increases in self-compassion and greater decreases in shame and fears of self-compassion, compared to waitlist controls (*n* = 20) [80]. This was a feasibility study examining the acceptability of a brief, internet-delivered self-compassion intervention. The intervention appeared to be acceptable and feasible, but changes in eating pathology (EDE-Q scores) and readiness to get help for one’s weight did not differ between conditions.

#### Manual-based cognitive remediation therapy (CRT)

##### Emerging adults

One qualitative study found that a CRT self-help manual and diary entries for individuals with AN and EDNOS (*n* = 6) were well-received according to high levels of satisfaction and acceptability reported by participants [81]. Both individuals with AN and EDNOS and parent participants commented that they would recommend the treatment to others; however, participants suggested that CRT be adapted for delivery via a computer rather than a manual.

#### Motivational enhancement treatment (MET) and self-help book

##### Emerging adults

One RCT with individuals with BN and BED compared a self-help only intervention (*n* = 45), consisting of a CBT-based book (‘Overcoming Binge Eating’) completed at the participants’ own pace, to in-person MET sessions supplemented with the same CBT-based book (*n* = 45) [82]. The MET intervention resulted in increased readiness to change for binge eating and significantly more participants in the MET condition were abstinent from bingeing at follow-up, compared with the self-help only intervention, but otherwise there were few differences between conditions for eating attitudes and frequency of binge eating and compensatory behaviours. With regard to eating behaviours, participants in both conditions had reduced frequencies of binge eating and compensatory behaviours, but within-group effects indicated that individuals in the MET condition experienced significant reductions in binge eating, compensatory behaviours, and maladaptive attitudes, whereas changes in the self-help only condition were not significant [82].

### E-technology as adjunctive interventions

#### CBT-based group internet interventions

##### Emerging adults

Two RCTs studying a CBT-based group internet intervention (‘Set your body free’ with eight weekly sessions led by a therapist) for emerging adults with probable BN and/or high body dissatisfaction were found [83, 84]. The pilot RCT compared face-to-face delivery (*n* = 19) versus internet-delivery (*n* = 21) modes of the program, reporting on feasibility [83], whereas the full RCT compared effectiveness of face-to-face delivery (*n* = 42), internet-delivery (*n* = 37), and delayed treatment control (*n* = 37) [84]. Both studies revealed large improvements in body dissatisfaction (Body Satisfaction Questionnaire [BSQ], Body Image Avoidance Questionnaire [BIAQ] scores) and dietary restraint (Dutch Eating Behaviour Questionnaire Restraint Scale [DEBQ-R] scores) in face-to-face and internet-delivery groups. In the pilot RCT, no significant differences between delivery modes were observed at post-treatment and at 2-month follow-up [83]. However, in the full RCT, post-treatment improvements were greater in the face-to-face than the internet intervention, but, generally gains made in both groups were no longer clearly different from each other at 6-month follow-up [84] (Table 6).

**Table 6:** CBT-based group internet interventions for emerging adults

Certainty assessment							Impact	Certainty	Importance
№ of studies	Study design	Risk of bias	Inconsistency	Indirectness	Imprecision	Other considerations			
**Outcomes: Body satisfaction, body attitudes, shape concerns, dietary restraint (BSQ, BIAQ, DEBQ-R)**									
2	randomized trials	not serious	not serious	not serious	not serious	none	2 RCTs for CBT-based group therapy with probable BN and high body dissatisfaction participants (in 1 pilot RCT: *n* = 19 face-to-face delivery and *n* = 21 internet-delivery [83]; in other RCT: *n* = 42 face-to-face delivery, *n* = 37 internet delivery, and *n* = 37 delayed treatment control [84]). Both face-to-face and internet delivery intervention groups showed large improvements in body dissatisfaction (BSQ, BIAQ scores) and dietary restraint (DEBQ-R). In the pilot study [83], no significant differences were found between face-to-face and internet-delivery modes at post-treatment and follow-up. In the full study [84], gains at post-treatment in face-to-face condition were greater than those in the internet group, but at 6-month follow-up generally gains made in both groups were no longer clearly different from each other.	⨁⨁⨁⨁ HIGH	CRITICAL

Bibliography:

RCTs – Gollings 2006 [83], Paxton 2007 [84]

#### Moderated online forums

##### Children/adolescents

One qualitative study examining a moderated online discussion forum (*n* = 119 users), which aimed to facilitate support between adolescents regarding their ED and the recovery process, determined several themes illustrating how young people use the forum [85]. Themes included taking on the role of the mentor, establishing a safe space online, forming friendships, acquiring help when needed, and seeking peer support for recovery and relapse prevention. The study concluded that moderated online discussions may foster a supportive environment in recovery for children and adolescents with an ED.

##### Emerging adults

One cross-sectional study found that a moderated online forum enabled empowerment for emerging adults experiencing ED symptoms through exchange of information and sharing experiences with others [86]. The most pronounced empowering outcome of using the forum was feeling better informed, and to a lesser degree, it increased help-seeking behaviour, optimism, control over the future, confidence in treatment; perceived improvements in the relationships with their therapists. The study determined that forum users had potential to become an active partner in the management of their ED.

#### Smartphone applications

##### Children/adolescents

One qualitative study compared the impact of TCApp between individuals with AN, BN, and EDNOS (*n* = 9), mobile health experts (*n* = 11), health care professionals (*n* = 10), and ED specialists (*n* = 8) [87]. TCApp is a mobile health app that connects children and adolescents with EDs with their therapists in the periods between medical consultation. The study found that most health care professionals considered the app difficult to use, with barriers related to external factors (e.g. lack of time because of workload), while individuals with EDs and ED specialists perceived the app as easy to use. Some individuals with EDs reported barriers related to use of the app including lack of personalization and motivational components, where they also expressed a lack of enthusiasm about the web-based chat option with ED specialists, although this was a facilitator for use from the perspective of ED specialists.

One mixed methods study asked children and adolescents meeting clinical or subclinical criteria for AN, BN, or BED (*n* = 366) about their interest in trying a hypothetical evidence-based mobile mental health app for EDs that included e-coaching [88]. Respondents with more severe manifestations of illness were more interested in trying the app, compared to those with less severe signs of an ED. Unwillingness to try the app was related to privacy concerns, worries of parents’ reaction, and feelings that their parents might not want them to participate.

##### Emerging adults

A variety of evidence was found related to smartphone apps for emerging adults with EDs. One RCT consisted of individuals with AN in a ‘Recovery Record’ plus treatment as usual intervention group (*n* = 20) versus a treatment as usual control group (*n* = 20) [89]. This was a feasibility study, examining the acceptability and preliminary effects of an innovative therapist-guided smartphone-based aftercare intervention following inpatient treatment. Recovery Record is a CBT-based mobile app which involves self-monitoring, encouraging feedback, and coping strategies, with a linking feature with the treating clinician enabling individuals with EDs to share self-entered data with their clinician. At post-intervention, this RCT found non-significant small to moderate between-group effect sizes favouring the smartphone app intervention group over the treatment as usual group regarding ED psychopathology (EDE-Q scores) and BMI. However, at the 6-month follow-up, there were no significant differences between intervention and control groups for these measures (Table 7).

**Table 7:** Smartphone apps for emerging adults

Certainty assessment							Impact	Certainty	Importance
№ of studies	Study design	Risk of bias	Inconsistency	Indirectness	Imprecision	Other considerations			
**Outcomes: ED psychopathology (EDE-Q); BMI**									
1	randomized trials	not serious	not serious	not serious	not serious	none	1 pilot RCT with individuals with AN (*n* = 20 in Recovery Record interventio*n* = guided CBT-based smartphone app+ treatment as usual; *n* = treatment as usual) [89]. At post-intervention, non-significant small to moderate between-group effect sizes favoured the intervention group regarding ED psychopathology (restraint and shape concerns, assessed from EDE-Q). At 6-month follow-up, effects wore off and no significant differences between the intervention and control groups were found.	⨁⨁⨁⨁ HIGH	CRITICAL
							At post-intervention, non-significant small to moderate between-group effect sizes favoured the intervention group regarding BMI. At 6-month follow-up, effects wore off and no significant differences between the intervention and control groups were found [89].		
**Outcomes: ED behaviours (restrictive eating, binge eating, compensatory measures)**									
1	open trial	very serious^a,b^	not serious	not serious	not serious	none	1 open trial of university students with clinical or subclinical EDs, excluding AN (*n* = 13 universities) [90]. Of the students that screened for an ED and enrolled in the Student Bodies-ED mobile app intervention, ED behaviours such as restrictive eating and binge eating significantly decreased over the course of the users’ time in the 3-year period. While vomiting and diet pill/laxative use were not found to significantly decrease among users during the 3-year period, reports of these types of ED behaviours were very low.	⨁⨁◌◌ LOW	CRITICAL

^a^No control condition

^b^No randomization

Bibliography:

RCT – Neumayr 2019 [89]

Open trial – Fitzsimmons-Craft 2019 [90]

In terms of non-randomized evidence, one case report saw 108,996 downloads of the Recovery Record app over a two-year period, and of 2503 ratings of acceptability, 84% rated the app as 5/5 [91]. From the case report, approximately 50% of Recovery Record app users stated that they do not currently receive ED treatment, suggesting that the app could be effective in reaching an underserved population. One mixed methods study consisting of nine individuals with severe body image and disordered eating concerns determined that a different CBT-based mobile app (‘Students Bodies – ED mobile app’) offering one-on-one in-app and phone-based coaching in addition to the app’s core sessions was rated as highly usable by participants [92]. This was a feasibility study, examining usability and engagement aspects of the app and virtual program. The average usability score was originally 78/100, but once modifications were made to the app based on user recommendations this score increased to 83/100. Furthermore, an open trial with 13 universities found encouraging results for the Student Bodies – ED mobile app. Specifically, participant restrictive eating and binge eating significantly decreased over the course of users’ time in the intervention. Vomiting and diet pill/laxative use were not found to significantly decrease over the course of the intervention, but reports of these behaviours were very low [90].

#### Text messaging

##### Emerging adults

There was conflicting evidence for text messaging interventions for emerging adults with EDs. In one open trial, 12 individuals with AN, subclinical AN, or BN received personalized, motivational text messages following in-person psychoeducation sessions, which were sent prior to participants’ mealtime [93]. This was a feasibility study, examining whether motivational text messages were acceptable as a CBT adjunct, and whether the text messages had an effect on behavioural outcomes. While the text messaging adjunctive therapy was deemed acceptable and feasible by participants in the open trial, there was no impact on behavioural outcomes including dietary restraint and kilocalorie intake, and underweight participants reported an increased desire to restrict in response to the text messages. In contrast, a case report with two individuals with BN found that a weekly text-messaging based intervention following discharge from inpatient treatment resulted in positive outcomes, including no binge eating or purging reported over the course of 14 weeks of use [94]. The results suggested that the use of text messaging aftercare offers the possibility of supplementing traditional psychotherapeutic treatments.

#### Email and/or online counselling

##### Children/adolescents

Two case reports described individuals with AN (total *n* = 3) used email as an adjunct to treatment to relay their mood, calorie intake, and ED behaviours to their treating physician [95, 96]. In both case reports, individuals generally described email as a positive treatment adjunct, allowing for increased contact with the physician and individuals becoming more aware of their ED behaviours by documenting them online. Weight gain was achieved by one of the two individuals in one case report (16-year-old female) [95]. One qualitative study with individuals with any type of ED (*n* = 4) using an online email counselling service reported that the service provided a valuable place for young people to gain additional support and reduce feelings of isolation [97].

##### Emerging adults

Two case reports with individuals with AN or BN (total *n* = 4) found that treatment supplemented with email between the individual and clinician benefitted individuals in terms of improving coping behaviours and creating a greater sense of trust with their therapist [98, 99]. Another case report described the effects of a clinician matching two groups of two individuals with AN (total *n* = 4) with similar ED struggles and arranged for them to communicate via email as a means of supporting each other (without involving the clinician) [100]. These participants reported positive feelings towards the email matching program, and according to their treating clinician, all progressed well in their treatment. Two qualitative studies with individuals with AN, BN and EDNOS (total *n* = 309) found email online counselling was rated positively amongst users, with the supportive comments, fast and easy contact, counsellors’ competence related to EDs, and the service being free of charge as some of the main facilitators for using the online service [101, 102]. Two cross-sectional studies [103, 104] and one mixed methods study [105] were related to the same online consulting service, which involved anonymous emails with a health care professional for free via a website. All three studies found that the service was often the initial point of contact between users with AN, BN, and BED and a professional to get help for their ED; relatives of those with EDs were also accessing the online consulting service to learn more about EDs [103–105]. Finally, one open trial with individuals with BN, BED, and EDNOS (*n* = 23) further described the impact of using email to communicate food intake and symptoms with a therapist and in return, receiving CBT-based or eclectic support from the therapist [106]. This pilot study examined the feasibility of recruiting patients using the internet (email), and whether therapy for BN can be delivered via email. Results indicated significant improvements in depressive and bulimic symptoms at 3-month follow-up [106].

### Caregiver interventions focused on child outcomes

#### Online guided parental self-help – FBT

##### Children/adolescents

One case series with 19 families found that the individuals with AN in the online guided parental self-help FBT intervention experienced weight gain similar to clinician-delivered FBT programs, with improvements in ED-related psychopathology (EDE-Q scores) also reported by the end of the treatment [107]. In this study, parents watched a series of pre-recorded videos and met with a therapist by phone or videoconference for 20–30 min once weekly. Using similar methodology, individuals in one open trial (*n* = 12 diagnosed with AN, *n* = 12 at risk for AN, and *n* = 22 at high-risk for AN) remained stable or increased in ideal body weight by post-intervention [108] (Table 8). This pilot study examined the feasibility, acceptability, and short-term efficacy of the program.

**Table 8:** Online guided parental self-help – FBT for caregivers of children/adolescents

Certainty assessment							Impact	Certainty	Importance
№ of studies	Study design	Risk of bias	Inconsistency	Indirectness	Imprecision	Other considerations			
**Outcomes: Weight gain; EDE-Q**									
1	case series	very serious^a,b^	not serious	not serious	not serious	strong association^c^	1 case series with 19 families caring for adolescents with AN. At the end of the treatment (guided caregiver FBT training) and at follow-up, the adolescents experienced weight gain similar to clinician delivered FBT programs (large effect size) [107].	⨁⨁⨁◌ MODERATE	CRITICAL
							ED-related psychopathology (EDE-Q scores) for those with AN improved by the end of the treatment [107].		
1	open trial	very serious^a,b^	not serious	not serious	not serious	none	1 pilot open trial with adolescents with, at risk, or at high-risk for AN (*n* = 12 diagnosed with AN, *n* = 12 at risk for AN, *n* = 22 at high risk for AN) [108]. At the end of the 6 family-based early intervention online sessions, adolescents remained stable or increased in ideal body weight (weight gain).	⨁⨁◌◌ LOW	CRITICAL

^a^No control condition

^b^No randomization

^c^Large effect size from baseline to end of treatment and baseline to follow-up [107]

Bibliography:

Case series – Lock 2017 [107]

Open trial – Jones 2012 [108]

### Caregiver interventions focused on caregiver outcomes

#### Unguided caregiver self-help using web-based and /or pre-recorded videos focused on psychoeducation and communication skills

##### Children/adolescents

One mixed methods study that involved a Meal Support Training program (pre-recorded videos and an accompanying manual) was well-received by families with a child with AN, BN or EDNOS (*n* = 40) [109]. Caregivers reported that the program was informative, convenient, and useful in fostering caregiver understanding and patience with their child and the ED recovery process (Table 9).

**Table 9:** Virtual parent meal support training for caregivers of children/adolescents

Certainty assessment							Impact	Certainty	Importance
№ of studies	Study design	Risk of bias	Inconsistency	Indirectness	Imprecision	Other considerations			
**Outcomes: Helpfulness of the contents of the video and manual for meal support training**									
1	mixed methods	very serious^a,b^	not serious	not serious	not serious	none	1 mixed methods study with 40 families caring for a person with AN, BN, or EDNOS [109]. The Meal Support Training program was reported as informative, convenient, and was well-received by families. Many caregivers reported that the manual and video resources helped them be more understanding and patient with their child and the recovery process.	⨁⨁◌◌ LOW	CRITICAL

^a^No control condition

^b^No randomization

Bibliography:

Mixed methods – Cairns 2007 [109]

##### Emerging adults

Two RCTs had mixed evidence for effectiveness measured by caregiver outcomes [110, 111]. One RCT that compared a web-based group (*n* = 23) and an in-person workshop group (*n* = 27) for caregivers of emerging adults with AN and BN revealed improvements in the Caregiver Accommodation and Enabling Scale for EDs favouring the web-based intervention, while changes in caregiver burden favoured the workshop [110]. This was a feasibility study, examining the efficacy and feasibility of a web- and workshop-based psychoeducational intervention for caregivers of people with EDs. Another RCT with caregivers of emerging adults with AN, BN, atypical AN, atypical BN, and EDNOS that compared a DVD intervention (*n* = 147) to a control condition (*n* = 138) found Caregiver Accommodation and Enabling Scales for EDs were not reduced by the DVD intervention, but caregiver burden was reduced by the intervention compared to controls [111] (Table 10).

**Table 10:** Unguided caregiver self-help using web-based and/or pre-recorded videos focused on psychoeducation and communication skills for caregivers of emerging adults

Certainty assessment							Impact	Certainty	Importance
№ of studies	Study design	Risk of bias	Inconsistency	Indirectness	Imprecision	Other considerations			
**Outcomes: accommodation and enabling scale for EDs; caregiver burden**									
1	randomized trial	not serious	not serious	not serious	not serious	none	1 feasibility RCT with caregivers for AN and BN emerging adults (*n* = 23 caregivers in web-based intervention; *n* = 27 caregivers in workshop intervention) [110]. Positive experiences were reported in both interventions. From baseline to end of intervention, small between-group effect sizes were observed for changes in Caregiver Accommodation and Enabling Scale for EDs, favouring the web-based intervention.	⨁⨁⨁⨁ HIGH	CRITICAL
							Changes in caregiver burden favoured the workshop intervention rather than the web-based intervention [110].		
1	randomized trial	not serious	not serious	not serious	not serious	none	1 RCT with caregivers for AN, atypical AN, BN, atypical BN, and EDNOS emerging adults (*n* = 147 DVD video training intervention; *n* = 138 control) [111]. Caregivers’ accommodating behaviours (accommodation and enabling scale for EDs) were not reduced by the DVD intervention.	⨁⨁⨁⨁ HIGH	CRITICAL
							Caregivers’ burden and psychological distress were more reduced by the DVD intervention than control [111].		

Bibliography:

RCTs – Dimitropoulos 2019 [110], Quadflieg 2017 [111]

#### Guided caregiver self-help – skills

##### Children/adolescents

One mixed methods study, consisting of 16 caregivers for individuals with AN or BN watching DVDs on ED care with supplemental telephone coaching, found caregiver general distress (measured by General Health Questionnaires scores) decreased significantly from baseline to post-intervention [112]. This was a pilot study, examining the feasibility and acceptability of this skills-based training for caregivers of people with EDs, and whether the anxiety, depression, and expressed emotion in caregivers were reduced. Caregivers also expressed high levels of satisfaction with most aspects of the intervention and reported improvements in psychological distress and depression following the intervention, although these measures did not reach statistical significance (Table 11).

**Table 11:** Guided caregiver self-help – Skills for caregivers of children and adolescents

Certainty assessment							Impact	Certainty	Importance
№ of studies	Study design	Risk of bias	Inconsistency	Indirectness	Imprecision	Other considerations			
**Outcomes: Caregiver general distress (General Health Questionnaire)**									
1	mixed methods	very serious^a,b^	not serious	not serious	not serious	none	1 pilot mixed methods study with caregivers (*n* = 16) for children with AN and BN [112]. Caregivers expressed high levels of satisfaction with most aspects of the DVD and coaching skills training. From baseline to post-intervention, caregiver general distress (General Health Questionnaire scores) decreased significantly.	⨁⨁◌◌ LOW	CRITICAL

^a^No control condition

^b^No randomization

Bibliography:

Mixed methods – Sepulveda 2008 [112]

#### Guided parental self-help CBT (skills training approach + workbook or CBT-based online modules)

##### Emerging adults

Three RCTs studying variations in guided caregiver self-help based in CBT reported somewhat similar findings in terms of expressed emotion and ED symptom impact scale for caregivers of emerging adults with AN [79, 113, 114]. One RCT found its skills training intervention group (‘Experienced Carers Helping Others’ [ECHO]; *n* = 134) had reduced caregiver expressed emotion levels and ED symptom impact scale scores, compared to the control group (*n* = 134) [79]. Another RCT found a CBT-based online module intervention (‘Overcoming Anorexia Online’; *n* = 33) significantly reduced caregivers’ anxiety and depression at post-treatment compared to a telephone and email hotline support control group (*n* = 30) [113]. This was a pilot study, examining the efficacy of a novel, web-based systemic CBT intervention for caregivers of people with AN. Other outcomes, including caregivers’ expressed emotion and ED symptom impact scores, also had greater reductions in the intervention group than the control group, however these were not statistically significant [113]. The third RCT compared a CBT-based online module intervention (‘Overcoming Anorexia Online’) plus professional guidance via telephone (*n* = 19) to a group that received the ‘Overcoming Anorexia Online’ intervention alone (*n* = 18), and reported similar reductions in caregiver expression of emotion and ED symptom impact scale scores between both groups, but no significant difference between groups [114] (Table 12). This was a feasibility study, examining the usefulness of an online information and skills development intervention for caregivers of individuals with AN.

**Table 12:** Guided parental self-help CBT (skills training approach + workbook or CBT-based online modules) for caregivers of emerging adults

Certainty assessment							Impact	Certainty	Importance
№ of studies	Study design	Risk of bias	Inconsistency	Indirectness	Imprecision	Other considerations			
**Outcomes: Expressed emotion scale; ED symptom impact scale**									
1	randomised trials	not serious	not serious	not serious	not serious	none	1 RCT with caregivers (*n* = 134 in telephone coaching ECHO intervention; *n* = 134 control) for individuals with AN [79]. Caregivers in the intervention group had reduced expressed emotion levels at patient discharge and 6-month follow-up.	⨁⨁⨁⨁ HIGH	CRITICAL
							Caregivers in the telephone coaching intervention group experienced greater reductions in ED symptom impact scale scores than in the control group [79].		
1	randomised trials	not serious	not serious	not serious	not serious	none	1 pilot RCT with caregivers for individuals with AN (*n* = 33 in OAO web-based intervention; *n* = 30 in telephone and email hotline control) [113]. Compared with control, the OAO intervention significantly reduced anxiety and depression in caregivers at post-treatment; caregivers in the OAO intervention had greater reductions in expressed emotion than controls, but these were not significant.	⨁⨁⨁⨁ HIGH	CRITICAL
							Similar to expressed emotion, caregivers’ ED symptom impact scale scores were also reduced across both groups, but there was no significant difference between groups [113].		
1	randomised trials	not serious	not serious	not serious	not serious	none	1 feasibility RCT with caregivers for individuals with AN (*n* = 19 OAO + guidance intervention; *n* = 18 OAO only) [114]. Levels of expressed emotion reported by caregivers at post-intervention were reduced but did not differ significantly between the groups. Those with AN did not perceive that their caregivers’ levels of expressed emotion had significantly changed.	⨁⨁⨁⨁ HIGH	CRITICAL
							Caregivers’ ED symptom impact scale scores were also reduced across both groups, but there was no significant difference between groups [114].		

Bibliography:

RCTs – Schmidt 2017 [79], Grover 2011 [113], Hoyle 2013 [114]

#### Moderated online caregiver forums

##### Children/adolescents

Two non-randomized studies evaluated the use and impact of moderated online forums for caregivers of children with EDs, both describing online tools positively [115, 116]. In a qualitative study, five mothers explained that they use moderated blogs (available on the FEAST-Families Empowered and Supporting Treatment of Eating Disorders website) as a tool to foster social support alongside FBT sessions [115]. One open trial that consisted of 13 caregivers with a child with AN engaging in weekly therapist-guided virtual chat sessions had high satisfaction ratings (91.7%) in addition to reports that the sessions were accessible, convenient, and easy to use [116]. This was a pilot study, examining the technical feasibility and acceptability of a therapist-guided, internet-based chat support group for caregivers involved in FBT for adolescent EDs. Caregivers also reported looking forward to the chat sessions as they assisted in coping with their child’s ED, and that they would recommend the chat to others [116] (Table 13).

**Table 13:** Moderated online caregiver forums and teleconferenced caregiver support groups for caregivers of children/adolescents and emerging adults

Certainty assessment							Impact	Certainty	Importance
№ of studies	Study design	Risk of bias	Inconsistency	Indirectness	Imprecision	Other considerations			
**Outcomes: Feasibility and acceptability**									
1	open trial	very serious^a,b^	not serious	not serious	not serious	none	1 pilot open trial with 13 caregivers with a child with AN [116]. The virtual chat room sessions led by an FBT-trained therapist had high satisfaction ratings (91.7%; feasibility and acceptability) among caregivers. Caregivers reported looking forward to the chat sessions and that they were accessible, convenient, and easy to use.	⨁⨁◌◌ LOW	CRITICAL
1	case report	very serious^a,b^		not serious	not serious	none	1 case report with 6 caregivers with a child with an ED, participating in teleconferencing group sessions to foster caregiver support [117]. Monthly in-person meetings were added as more participants became interested. Overall, caregivers in the teleconferencing support groups felt they were helpful for those who feel emotionally isolated (feasibility and acceptability), but in-person meetings for support may be more ideal.	⨁⨁◌◌ LOW	CRITICAL

^a^No control condition

^b^No randomization

Bibliography:

Open trial – Binford Hopf 2013 [116]

Case report – Marx 2006 [117]

##### Children/adolescents and emerging adults

A case report using teleconferencing for caregiver (*n* = 6 parents) support for any ED in youth between the ages of 12 and 22 found this approach may be promising for developing healthy caregiver coping mechanisms during their child’s recovery [117]. However, these authors concluded that in-person meetings may be more ideal (Table 13).

### In-person care versus home monitoring

#### In-person medical evaluation

##### Emerging adults

Some evidence was found describing factors to consider when deciding if individuals with EDs should be seen in-person for evaluation following their remote care in accordance with COVID-19 social distancing regulations. A descriptive study indicated that a person with an ED should be asked to come in to a clinic for limited in-person sessions if they were clearly engaging in concerning behaviours (e.g. increased restricting, purging, over exercising) according to self or caregiver reports or if the provider or caregiver had a high index of suspicion for medical deterioration [47]. Furthermore, a commentary described that in the COVID-19 context, ED programs utilized weight and heart rate criteria to define urgency and need for in-person medical assessments, either from temporary ‘vital signs clinics’ where individuals with EDs would come in to a clinic for daily heart rate, blood pressure, and weight assessment (that was either followed by or preceded by a telehealth visit), or some programs enlisted parents to monitor and report vital signs including teaching parents to take pulses and assessing weights on home scales [48].

#### Home monitoring

##### Children/adolescents

There was some limited evidence from a pilot open trial assessing the feasibility of wearable sensors combined with wireless technologies for children and adolescents with AN (*n* = 27) to monitor heart rate and heart rate variability by a clinician (not by caregivers) in a remote setting [118] (Table 14). However, other recent evidence described interventions for home monitoring to be performed by caregivers of children with EDs during the COVID-19 pandemic. For example, two descriptive studies discussed home monitoring for children and adolescents with AN in which caregivers were enlisted to measure their child’s weight, heart rate, and blood pressure (Table 14), as well as record food intake and estimate energy expenditure, and report all findings to the clinician during telehealth sessions; clinicians were to visually assess individuals with EDs during telehealth visits [44, 46]. Overall, these new responsibilities for caregivers may contribute to a higher caregiver burden. Other research suggests that only weight be monitored by caregivers at home [26].

**Table 14:** In person care versus home monitoring for children and adolescents

Certainty assessment							Impact	Certainty	Importance
№ of studies	Study design	Risk of bias	Inconsistency	Indirectness	Imprecision	Other considerations			
**Outcomes: Vital signs (heart rate and heart rate variability)**									
1	open trial	very serious^a,b^	not serious	not serious	not serious	none	1 pilot open trial comparing adolescent girls with AN using wearable sensors plus wireless technologies to monitor heart rate and heart rate variability (*n* = 27) versus healthy controls (*n* = 15) [118]. Results showed that the wearable sensors were feasible to monitor vital signs in adolescents with AN, but by a clinician rather than a caregiver.	⨁⨁◌◌ LOW	CRITICAL
**Outcomes: Monitoring weight at home**									
1	case report in a descriptive study	very serious^a,b^	not serious	not serious	not serious	none	1 case report in a descriptive study with 1 child with AN [44]. The family utilized telehealth for FBT during COVID-19 where the child’s caregivers successfully measured the child’s weight at home using a home scale, as well as blood pressure and heart rate and reported these measures to the clinician.	⨁⨁◌◌ LOW	CRITICAL

^a^No control condition

^b^No randomization

Bibliography:

Open trial – Billeci 2015 [118]

Case report – Wood 2020 [44]

### Sex, gender, and diversity considerations

There was no evidence found related to the impact of sex, gender, and other considerations on virtual care for children, adolescents or emerging adults.

## Recommendations

### Section 1. Telehealth using synchronous videoconferencing and/or teleconferencing

#### Telehealth FBT may be a reasonable treatment option for *children and adolescents with AN*

#### Weak recommendation

##### ***Qualifying Statements:***

Family-Based Treatment (FBT) focuses on empowering parents to renourish their children. Although the evidence is rather scant, the panel member clinicians indicated that due to COVID-19 many of them are now using FBT by telehealth in their routine clinical practice and they suggest that there is good clinical reason to support these methods. Although these are treatment adaptations to a virtual mode of delivery, this treatment is known to be effective in-person, which adds confidence that it is likely beneficial when delivered virtually as well. There is urgency to adopt these treatments and likely minimal harm involved in delivering them by telehealth, as long as individuals are properly monitored medically. Some clinicians have been reluctant to have parents weigh their own children which would be a necessary component of FBT by telehealth.

***Key Evidence:***

- Significant weight gain from baseline to end of treatment and/or at follow-up was seen among individuals with AN and atypical AN in one feasibility open trial (with moderate to large effect sizes; *n* = 10) [56] and in one case report (*n* = 1) [57]. In the open trial videoconferencing was using to deliver FBT and in the case report, the telephone was used.

#### Telehealth relapse prevention using MANTRA may be a reasonable treatment option for *emerging adults* with AN

#### Weak recommendation

##### ***Qualifying Statements:***

Maudsley Model of AN Treatment for Adults (MANTRA) aims to address the cognitive, emotional, relational and biological factors which tend to maintain AN. This study followed inpatient admission/day patient treatment for AN. Eight sessions were delivered by videoconference; the first and last sessions were in person. Although this was an open trial, this intervention could be quite useful for those leaving intensive treatment and could provide a bridge to outpatient care.

***Key Evidence:***

- At the end of treatment (4 months, 10 sessions), a pilot open trial with individuals with AN (*n* = 16) resulted in increased BMI and reduced eating, shape, and weight concerns (EDE-Q scores) among participants [58].

##### Additional promising interventions

- Telehealth cognitive and behavioural treatments for ARFID and OSFED require more study [59, 60]. Although there is currently no evidence for family interventions for OSFED and ARFID delivered by telehealth, this should also be a focus of research.

### Section 2. Self-help and guided self-help

#### Internet CBT-based guided self-help is strongly recommended for *emerging adults* with AN, BN, BED, and EDNOS, as well as relapse prevention in AN

#### Strong recommendation

##### ***Qualifying Statements:***

There is strong evidence that internet CBT-based guided self-help is effective for emerging adults with AN, BN, BED, and EDNOS, and possibly for relapse prevention in AN. These young adults likely need to be motivated for recovery in order to benefit. Although CBT-based bibliotherapy had slightly less evidence (compared to CBT-based internet therapy), there was still some evidence that it was beneficial. The panel emphasized that many parents request psychoeducation on EDs, so there was endorsement of bibliotherapy not only for individuals but for parents as well. The panel recognized that books and workbooks on EDs may be especially useful if no internet or computer is available. One pitfall to all of these treatments is that speaking and reading in English is required, since all of the treatments included in this current review required speaking and reading in English, but it should be noted that translations are becoming available for some books on EDs.

***Key Evidence:***

CBT – Internet Guided Self-Help

- One RCT with those self-reporting AN, BN, BED, or EDNOS symptoms, assessed by the Short Evaluation of Eating Disorders (SEED): total *n* = 87 Featback, *n* = 88 Featback + low-intensity therapist support, *n* = 89 Featback + high-intensity therapist support; *n* = 90 waitlist control. Interventions were superior to control in reducing ED psychopathology (SEED and EDE-Q scores). No added value of therapist support in symptom reduction but contributed to greater satisfaction. No significant differences between therapist support conditions. Lowest costs in condition with low-intensity therapist support [61].
- One RCT with those diagnosed with BN or EDNOS: *n* = 38 ‘Overcoming Bulimia Online’ intervention; *n* = 38 waitlist control. Intervention group had higher rates of cessation from binge eating and purging than delayed treatment condition; gains maintained or continued to improve at follow-up [62].
- Relapse prevention in AN – one RCT: VIA intervention (relapse prevention based in CBT after AN inpatient treatment) *n* = 128, control *n* = 130. Moderate dropout rate (15.5%); intervention completers gained significantly more weight (individuals with AN) than controls; favourable course for BMI-adherence to intervention, more spontaneity, better self-esteem [63].

CBT-based Bibliotherapy

- One RCT with those diagnosed with BN or EDNOS (with bulimic symptoms): total *n* = 70 internet guided self-help (INT-GSH); *n* = 56 bibliotherapy guided self-help (BIB-GSH). ED symptoms (binge eating and compensatory behaviour) improved significantly in both groups (no significant differences regarding outcomes between groups). No group differences in Eating Disorder Inventory (EDI) subscales (adolescents *n* = 29, adults = 97) [75].

#### Internet-based relapse prevention MANTRA guided self-help may be a reasonable treatment option for *emerging adults* with AN

#### Weak recommendation

##### ***Qualifying Statements:***

Internet-based Maudsley Model of AN Treatment for Adults (iMANTRA) involved a workbook and email support by a therapist 1–3 times a week for the first 6 months, then 1 time per week for months 7–12. Workbook content included nutrition planning, coping strategies, and strategies to reduce anxiety. This relapse prevention feasibility study examined the use of email guided self-care treatment added to treatment as usual post hospital or day treatment. A key aim of iMANTRA was to facilitate patient engagement in ongoing outpatient treatment, so the generalizability of this study to other populations is limited.

***Key Evidence:***

- One feasibility RCT with individuals with AN *n* = 24 iMANTRA intervention + treatment as usual; *n* = 17 treatment as usual. At 6 months, there was little difference between groups; at 12 months, the intervention group had a higher BMI and lower scores on Depression, Anxiety, and Stress Scale (DASS-21) than controls. Despite the differences between groups at 12 months, confidence intervals were wide and overlapped with zero, which decreases the certainty of the findings [79].

##### Additional promising interventions

- Internet-delivered self-compassionate letter writing unguided self-help for *emerging adults* with AN and atypical AN requires more study [80].

### Section 3. E-technology as adjunctive interventions

#### CBT-based group internet interventions may be a reasonable adjunctive treatment option for *emerging adults* with high body dissatisfaction

#### Weak recommendation

##### ***Qualifying Statements:***

Studies used a cognitive behavioural program called ‘Set your body free’. This program included eight weekly 90 min group sessions led by a therapist by synchronous internet delivery via a chat room and using a manual (6–8 participants per group). Participants had high degrees of body dissatisfaction and the authors indicate that they had probable BN, but it is difficult to make firm conclusions as a diagnosis of BN was not required to participate in the sessions. As a result, some participants in these studies may not have met criteria for BN, however, our recommendation suggests that those with BN would likely benefit. One issue with virtually delivered treatments is that the generation of a diagnosis is more challenging and sometimes a diagnostic interview is not completed. Although evidence suggests that the ‘Set your body free’ program may be useful for addressing body dissatisfaction and may be helpful for those with BN, this is not a standalone treatment for body dissatisfaction or BN.

***Key Evidence:***

- Two RCTs with those exhibiting BSQ scores above the community mean (> 81.5) and/or those who possibly had BN (according to BULIT-R scores): *n* = 19 face-to-face delivery and *n* = 21 internet-delivery [83]; *n* = 42 face-to-face delivery, *n* = 37 internet-delivery, and *n* = 37 delayed treatment control [84]. Both face-to-face and internet groups showed large improvements in body dissatisfaction (BSQ, BIAQ scores) and dietary restraint (DEBQ-R) (compared to control in the RCT with a delayed treatment control group); in the pilot RCT, no significant differences between delivery modes were observed at post-treatment and at 2-month follow-up [83]. However, in the full RCT, post-treatment improvements were greater in the face-to-face than the internet intervention, but generally gains made in both groups were no longer clearly different from each other at 6-month follow-up [84]; both were effective.

#### Guided CBT-based smartphone apps may be reasonable adjunctive treatment options for *emerging adults* with AN

#### Weak recommendation

##### ***Qualifying Statements:***

There was much discussion on the panel regarding the CBT app (Recovery Record) for emerging adults. The context in which the study was done was post inpatient admission for AN in emerging adults for a period of 8 weeks with feedback from a therapist on a weekly basis at minimum. Some panel members felt we should ensure the app was recommended only as an adjunct to standard treatment, offered concurrently. Some wondered if there could be unintended harms by individuals with EDs believing that they were in treatment by use of the app, when in fact they were not really making progress toward recovery, or that the app did not provide the level of treatment needed for those with more severe EDs. Some felt that these apps could serve a purpose in meeting young people where they are at in terms of level of motivation and that such an app might set them on a pathway to connect with more standard care. The app might also be able to reach more people who could not access standard care. We agreed to leave this as a weak recommendation, with the caveat that in more severe presentations of EDs, the app may be insufficient, and that the app should be used as it was in the study with guidance from a therapist. In fact, the panel felt it should only be endorsed as an adjunctive treatment, accompanying standard treatment.

***Key Evidence:***

- One pilot RCT with emerging adults diagnosed with AN: at post-intervention, non-significant small to moderate between-group effect sizes favoured the smartphone app intervention group with therapist feedback (received support from therapist through the app two times per week for 25 min each for the first 4 weeks, which decreased to once per week during the last 4 weeks; *n* = 20) regarding ED psychopathology (EDE-Q scores) and BMI vs. control (*n* = 20); at 6-month follow-up: no significant differences between intervention and control groups for these measures [89].

##### Additional promising treatments

- Email therapy (as a treatment adjunct) for children and adolescents with AN requires more study [96].
- Email therapy and/or online counselling (as a treatment adjunct) for emerging adults with AN, BN, BED, and EDNOS requires more study [98, 99, 105, 106].
- Text messaging therapy (as a treatment adjunct) for emerging adults with AN, subclinical AN, and BN requires more study [93, 94].

### Section 4. Caregiver interventions focused on child outcomes

#### Online guided parental self-help FBT may be a reasonable treatment option for *children and adolescents* with AN, and at risk for AN

#### Weak recommendation

##### ***Qualifying Statements:***

Research evidence evaluates the use of pre-recorded videos that parents can access via a web-based platform, along with coaching by a therapist via phone or videoconferencing. The videos focus on the principles of FBT including urgency to act, caregiver empowerment, externalization of the ED, agnosticism, and psychoeducation around medical complications. One study involved children and adolescents diagnosed with AN (according to DSM-5 criteria for AN), and the other included those with AN, at risk for AN, and at high risk for AN, as determined by a diagnosis of AN within the past 6 months and/or screening results using established risk factors for EDs, retrospective correlates, and/or early symptoms of AN.

***Key Evidence:***

Online guided parental self-help – FBT

- One case series: *n* = 19 families. At the end of the treatment and at follow-up, individuals with AN experienced weight gain similar to standard clinician-delivered FBT programs. ED-related psychopathology (EDE-Q scores) of the children improved by end of treatment [107].
- After the family-based early intervention sessions (six online sessions) using pre-recorded videos, individuals in one pilot open trial (*n* = 12 diagnosed with AN, *n* = 12 at risk for AN, and *n* = 22 at high risk for AN) remained stable or increased in ideal body weight [108].

### Section 5. Caregiver interventions focused on caregiver outcomes

#### Virtual parent meal support training may be beneficial for caregivers of *children and adolescents* with AN, BN, and OSFED

#### Weak recommendation

##### ***Qualifying Statements:***

Caregivers watched pre-recorded video content and receive a manual on meal support strategies.

***Key Evidence:***

- One mixed methods study: the virtual Meal Support Training program was well-received by families (*n* = 40; indicated by the lowest rating of the program being 3.79 [SD = 0.843] on a 5-point scale [1 = not informative, 3 = somewhat informative, 5 = very informative]), and families deemed the intervention as informative and convenient; caregivers reported that the program helped them to be more understanding and patient with their child and the ED recovery process [109].

#### Unguided caregiver self-help using web-based and/or pre-recorded videos focused on psychoeducation and communication skills, may be beneficial for caregivers of *emerging adults* with AN, atypical AN, BN, atypical BN, and EDNOS

#### Weak recommendation

##### ***Qualifying Statements:***

One study focused on a web-based intervention with 8 modules and a workbook. Core elements of the intervention were ED psychoeducation, communication skills, meal support strategies, caregiver needs. The other study provided 5 pre-recorded information sessions to caregivers on the impact of EDs, meal support strategies, communication skills, motivational stages of change. There was no contact with a therapist.

***Key Evidence:***

- One feasibility RCT with caregivers for individuals with AN and BN: *n* = 23 web-based group; *n* = 27 workshop group. Improvements in Caregiver Accommodation and Enabling Scale for EDs favored web-based intervention; changes in caregiver burden favored workshop intervention [110].
- One RCT with caregivers for individuals with AN, BN, atypical AN, atypical BN, and EDNOS: *n* = 147 DVD intervention; *n* = 138 control. Caregivers’ Accommodation and Enabling Scale for EDs scores were not reduced by DVD intervention, but caregiver burden was reduced by intervention compared to control [111].

#### Guided parental self-help CBT (skills training approach + workbook or CBT-based online modules) is strongly recommended for caregivers of *emerging adults* with AN, and may be effective for caregivers of children and adolescents with AN or BN

#### Strong recommendation

##### ***Qualifying Statements:***

One study used Experienced Carers Helping Others (ECHO)- guided self-help skills training for caregivers using a book, pre-recorded videos and telephone coaching sessions. Another two studies used a web intervention using interactive multimedia CBT for caregivers to help them understand and meet their own needs; the latter intervention was called Overcoming Anorexia Online (OAO). The study involving caregivers of children and adolescents used a series of pre-recorded videos on communication skills, cycle of change, and strategies for supporting eating. Telephone or email support was also provided by a clinician. The panel agreed that these interventions for caregivers should be used in addition to treatment for the individual with an ED and should not be confused with actual treatment.

***Key Evidence:***

Guided self-help for caregivers of emerging adults

- One RCT with caregivers for individuals with AN: skills training intervention (ECHO) (*n* = 134) had reduced caregivers’ expressed emotion levels and ED symptom impact scale scores**,** compared to controls (*n* = 134) [79].
- One feasibility RCT with caregivers for individuals with AN: Compared with telephone and email hotline support control (*n* = 30), the CBT-based online modules intervention group (OAO; *n* = 33) significantly reduced anxiety and depression in caregivers at post-treatment; caregivers in the OAO intervention group also had greater reductions in expressed emotion and ED symptom impact scale scores compared to controls, but these were not significant [113].
- One feasibility RCT with caregivers for individuals with AN: Caregiver expressed emotion and ED symptom impact scale scores were similarly reduced in both groups (*n* = 19 OAO + guidance; *n* = 18 OAO only); no significant difference between groups [114].

Guided caregiver self-help – Skills for caregivers of children and adolescents

- One pilot mixed methods study with caregiver for individuals with AN and BN (*n* = 16). From baseline to post-intervention, caregiver general distress (General Health Questionnaire scores) decreased significantly [112].

#### Moderated online caregiver forums and support groups may be beneficial for caregivers of *children and adolescents* with AN

#### Weak recommendation

##### ***Qualifying Statements:***

Although the evidence was minimal, the online forum for caregivers was given a weak recommendation due to the enormous benefit to parents, as well as popularity of the Maudsley Parents forum. A note to readers here is that we did not include the search term ‘social media’ in our database searches; the panel agreed a priori that the research related to social media would be too large to review and would not meet our goals of answering our research questions related to the best treatments that can be delivered virtually or in the COVID-19 context. Thus, we focused on moderated forums (as opposed to unmoderated forums). The panel mentioned the importance of parent support as helpful in reducing caregiver burden. From their own experience running virtual parent-led support groups, many parents would prefer in-person support, but are glad there is a virtual option currently being offered in some locations. Virtual options might have benefits in terms of time and being able to access from home (no need for caregivers to arrange childcare). It is important to note here that no evidence exists to date for parental forums having an impact on child and adolescent outcomes. Further study is needed.

***Key Evidence:***

- One pilot open trial with caregivers for individuals with AN: virtual chat room sessions were highly feasible and acceptable (91.7% satisfaction rating) among caregivers (*n* = 13), who reported sessions as accessible, convenient, and easy to use [116].
- One case series of teleconferencing for caregiver support for caregivers of those with any ED (not specified) aged 12–22 years found benefit for caregivers [117].

### Section 6. In person care versus home monitoring

#### It is strongly recommended that individuals (of all ages) with EDs are seen in-person for medical evaluation by specialists in conjunction with local care providers when necessary and that international guidelines and criteria for admission are followed

#### Strong recommendation

##### ***Qualifying Statements:***

This recommendation is based on expert consensus. The panel was unanimous in their agreement that it is not possible to offer all care virtually to this patient population. Guidelines by the Society for Adolescent Health and Medicine provide criteria for hospital admission and medical monitoring [119] in children, adolescents, and emerging adults and should be followed by all practitioners. Individuals with EDs must be able to access emergency rooms and hospital beds despite competing demands for resources in the COVID-19 era. The threshold for admission to hospital should not be changed due to the COVID-19 context. In fact, a direct relationship may exist between the pandemic and an increase in the number of young people needing admission to hospital for EDs. This was mentioned by panel members and there is preliminary evidence to support this (personal communication with several pediatricians across the country, and a study from Perth, Australia [120]).

In terms of monitoring at home, weight can be taken at home on a home scale, however FBT therapists should educate caregivers around possible falsification of weight. Monitoring of vital signs at home is not recommended due to difficulties in proper measurement interpretation and issues with methods of monitoring (may not be reliable or accurate) that could cause harm. Vital signs should be monitored by a health professional. Lack of progression in virtual therapy, concerns around accuracy of home weights, or new symptoms should signal the need for in-person evaluation.

Suicidality is another reason individuals may need to be assessed in-person. Should a young person not wish to come to hospital, but there is grave concern over physical or mental health, legal processes should be followed in order to ensure that the individual receives the assessment and treatment that they require.

***Key Evidence:***

- Some limited evidence of the feasibility of wearable sensors for adolescents with AN to monitor vital signs by a clinician, not by caregivers (*n* = 27) [118].
- Some studies indicate that with rapid scale up of virtual FBT, caregivers for those with AN were given the role of monitoring weight at home [44, 46].

### Section 7. Sex, gender, and diversity considerations

#### We strongly recommend that equity-seeking groups and marginalized youth should be provided equal access to treatment

#### Strong recommendation

##### ***Qualifying Statements:***

This recommendation was not based on research evidence, but rather on expert consensus. Equity-seeking groups as defined by the Public Service Alliance of Canada include racialized people, people with disabilities, Aboriginal (First Nations, Inuit and Métis), women, lesbian, gay, bi-sexual, and trans individuals [121]. Trans-youth and boys are at high risk of not being recognized by providers as having EDs. Providers need to be more vigilant with these groups, and also be aware of the barriers to care that they face. In addition, non-English speaking youth and their families struggle with extra barriers to care, especially in a virtual world. It is difficult for an interpreter to be involved virtually, although this should be attempted. Those in rural areas or of lower socioeconomic status may not have access to the internet, or they may face limitations related to internet speed and high cost. Providers should also be vigilant to violence occurring in homes as individuals experience heightened isolation. Health care workers should be aware of how racism affects the quality of and access to health care for racialized groups and should seek to reduce barriers. Indigenous peoples, especially in the far north, face additional barriers to care related to geographical isolation, lack of access to the internet or reliable internet, lack of access to videoconferencing centres, as well as lack of confidentiality due to small population sizes and therefore, increased likelihood of kinship, peer, or social connections to health care providers. Inclusion of equity-seeking groups in research is essential.

***Key Evidence:***

No evidence could be found in our search on these topics.

## Discussion

These are the first Canadian Practice Guidelines to evaluate the evidence on virtual care focused specifically on children and adolescents (< 18 years) and emerging adults (18–25 years) with EDs, in the COVID-19 context. A strong recommendation was supported in favour of in-person medical evaluation, when necessary, for children, adolescents, and emerging adults with EDs; as was a strong recommendation that equity-seeking groups and marginalized youth should be provided equal access to treatment. In addition, for emerging adults, internet CBT-based guided self-help for AN, BN, BED, and EDNOS was strongly recommended. Weak recommendations were generated for CBT-based group internet interventions as treatment adjuncts for high body dissatisfaction, internet-based relapse prevention MANTRA guided self-help for AN, telehealth relapse prevention using MANTRA for AN, and guided CBT-based smartphone apps as treatment adjuncts for AN in this age group. For children/adolescents with AN, weak recommendations were supported for telehealth FBT, and online guided parental self-help FBT. In terms of caregiver outcomes, guided parental self-help CBT for caregivers of emerging adults with AN or BN was strongly recommended, while unguided caregiver psychoeducation self-help was weakly recommended for most ED diagnoses. For caregivers of children and adolescents with EDs, weak recommendations were supported for virtual parent meal support training, as well as moderated online caregiver forums and support groups.

Recommendations were developed by taking into account research evidence, knowledge that treatments delivered in person generally translate well to virtual care, and the values, preferences, and opinions of panel members. In the absence of more robust research on the efficacy of virtually delivered ED treatments – particularly in the COVID-19 context – clinicians should use these guidelines and recommendations to inform their clinical decisions regarding what are likely the most effective and implementable treatments. Systematic review and meta-analysis evidence from non-ED research suggest that psychological treatments transfer well to virtual delivery, without the loss of efficacy or patient acceptability [12–14]**.** Although the research evidence reviewed in this guideline may be perceived as weak on its own, the fact that the effects of virtually-delivered ED care also seem to be comparable to in-person delivery reinforces these conclusions. Readers must consider the fact that the evidence presented in studies conducted prior to the pandemic is within a context in which individuals with EDs, caregivers, and clinicians opted into virtual care or were randomized to virtual care having consented to participation. In the COVID-19 context, the vast majority of individuals now delivering or receiving virtual treatment have been left without a choice but to do so. Once restrictions are lifted and there is a gradual return to face-to-face treatment, it is possible that virtual care may become less acceptable.

Best practices for virtual therapy have been previously published pertaining to non-ED-related care [122–125]. Generally, these considerations can be applied to virtual ED therapy and clinicians would benefit by their review. See Table 15 for good practice points related to virtual care, summarizing issues that clinicians from all fields should be mindful of when delivering treatments virtually.

**Table 15:** Good Practice Points to Consider for Virtual Care

**Practical/Technical Issues:** Practitioner and patient preference, experience level, and organizational capacity should be considered when choosing a virtual platform, but reliable, secure video-call platforms should be favoured over the use of audio calls (e.g. to conduct a visual assessment of a patient). Prior to commencing online sessions, clinicians should prepare and share with patients a written plan detailing what to do in the event of technological failure (e.g. who should first attempt to re-establish a lost connection, when to attempt alternate technologies, how long to wait before presuming that a connection cannot be re-established).	
**Inclusivity:** Virtual delivery poses a potential barrier to care for those without home internet access, or an electronic device, so alternatives such as books and workbooks for patients and caregivers should be encouraged where necessary. Language, sight/hearing impairment, and technological experience should also be considered (e.g. involving an interpreter in the care of non-English speaking individuals).	
**Confidentiality:** Lack of privacy in homes may impact patient engagement in virtual therapy; headphones or establishing a ‘time-out signal’ can help to increase the level of privacy during sessions. End-to-end encryption should be prioritized, and passwords should be used regularly. Clinicians should inform patients of the measures being taken to protect their privacy and security.	
**Managing Medical and Psychological Safeguarding Risks:** Clinicians providing virtual care must continue to practice within the jurisdiction in which they are licensed and according to their insurance coverage for malpractice. Crisis management plans should be available for every patient involved in virtual treatment. These plans can include patient-specific local resources (e.g. crisis lines, hospitals, emergency services). Clinicians must be able to coordinate care with local crisis resources if they are required (e.g. in case of high suicidality risk) and should be familiar with local mental health laws in responding to crises obligations (e.g. duty to call child protective services, when to call police). Creating a network with local providers may also be beneficial in the case of an emergency. Clinicians should also determine if the family context and home environment are considered safe and non-triggering for virtual contacts.	
**Managing Intensity, Difficult Family Dynamics, or Overwhelming Emotions:** Clinicians should be vigilant to any violence or maltreatment that may be occurring in the family home. Follow-up with participants of virtual sessions that become highly intense, and when necessary, referral to additional health resources, is encouraged.	
**Length of Sessions:** Duration of sessions may need to be changed, depending on the patient (e.g. briefer sessions may be more effective for younger patients/siblings with shorter attention spans).	
**Cultural and Socio-Economic Factors:** The needs of equity-seeking groups should be acknowledged before and during treatment and reducing barriers to care should be of high priority. Clinicians and patients engaging in virtual therapy from their respective homes may result in feelings of blurred boundaries. To counteract this, participants should be encouraged to create a particular space in the home for virtual care. Involvement of parents should be explicitly discussed, as it is important to devise a plan outlining how family members will be involved (e.g. reserving time, being present when needed, and clarifying roles in treatment).	

Panel members generally commented on the pros and cons of virtual care. While virtually delivered care may be easily accessible for those with a reliable internet connection at home, this poses a potential barrier to care for families without home internet access. Clinicians mentioned fatigue from virtual care and challenges navigating the legal and procedural aspects of technology-based care. Others mentioned that their patients and families often do not like to see their own image, which poses challenges when trying to conduct video call appointments. Suggestions for this problem were proposed by the panel including having the family block their own image by covering this portion of their own screen (e.g. hiding their image on the videoconferencing platform or using a sticky note on their monitor). Some panel members also indicated it is hard to evaluate weight status in patients by virtual means. This resonates with the literature already published on this topic. Three studies identified ED professionals’ opinions on the impact of digital technologies on their patients, indicating potential benefits and drawbacks. Specifically, in two qualitative studies (total *n* = 65) professionals described advantages in virtual care including better patient-clinician communication and access to patient reported data (via apps) ahead of scheduled sessions. However, disadvantages included added workload for the clinician and the risk that it may be easier for patients to continue with and hide their ED symptoms and behaviours in an online environment [126, 127]. Survey results from one cross-sectional study found that professionals (*n* = 282) believe e-health may be a more useful treatment for adolescents and adults than children and seniors, and may be more beneficial for anxiety and depressive disorders than for addiction and EDs [128].

An additional aspect to consider is how to appropriately train clinicians in virtual modalities. Although thought to be equally efficacious, virtual adaptations of treatments may have differences that could affect efficacy. Three studies observed the effectiveness of various web-centered ED training programs for health care professionals. In one RCT that compared a CBT-E web-centered supported training group (receiving phone call support in addition to the course components; *n* = 81) to a CBT-E web-centered independent training group (only received access to the CBT-E course; *n* = 75), it was confirmed that web-centered training can successfully train therapists to deliver CBT-E [129]. The supported and independent training programs were also equally effective as both training groups had increased scores in therapist competence from pre-intervention to post-intervention [129]. This CBT-E web-centered training program was also studied in an open trial (*n* = 765), similarly confirming that competence in delivering CBT-E was improved following web-based training. Factors associated with a beneficial effect from this training included compliance with training recommendations, higher number of training modules completed, and treating at least one patient while in training [130]. In a separate open trial that involved a tele-mentoring project between ED specialists (mentors) and community-based practitioners (mentees) (Project ECHO; *n* = 99) which aimed to disseminate specialized ED knowledge to clinicians in need and located in underserved areas via Zoom, tele-mentoring was found to be promising to bridge the speciality-based knowledge gap between ED-trained and front-line clinicians [131].

Panel members also discussed the various platforms available for virtual care delivery. There is limited evidence describing what online platform is the most ideal to deliver ED care virtually, and as such, clinicians tend to only use what is available through their institution. However, one descriptive study that summarized the opinions of clinicians delivering CBT for EDs via telehealth during COVID-19 revealed positive experiences about working with cloud-based videoconferencing and live-chat services, including Zoom, Facetalk, Google Meets, Vsee, and Microsoft Teams [50]. Other software choices, such as Skype and FaceTime were seen as less reliable and less secure in comparison, and therefore were not often recommended [50]. According to this descriptive study, platforms that allow for confidential sharing of documents during sessions, high security, white board feature, and ease of use may be the most favourable to support virtual care. However, other platforms endorsed by Canadian health care agencies as meeting privacy and security requirements were not evaluated in this particular study. Our panel also did not endorse one single platform; panel members identified benefits to several different platforms and acknowledge that practitioner context and organizational mandates may be a key factor in the selection of virtual technology.

Two studies described general recommendations for clinicians delivering virtual mental health interventions during COVID-19. One commentary focused on recommendations for improving the delivery of virtual mental health care, such as: programs offering training and supervision in virtual therapy tools, national licenses being implemented to practice virtual therapy (telehealth), providers being taught to ensure patient confidentially during virtual sessions, broadening reimbursement coverage to include evidence-based virtual therapies, continuous evaluation of the efficacy of virtual therapies on broader mental health disorders, and introducing innovative and timely virtual mental health practices into health care systems [132]. One descriptive review noted that guidelines for telepsychiatry in EDs are lacking, but in general, clinicians involved in telepsychiatry during COVID-19 should: a) familiarize themselves with the telehealth system to ensure sessions run smoothly; b) focus on effective communication during sessions; and c) document any issues with telehealth immediately, so that improvements can be made for future sessions [133].

### Strengths

The strengths of this guideline are numerous. We used a rigorous and evidence-based methodology for our scoping review and our guideline development. Our scoping review methods included a thorough review of all literature (including a variety of databases, grey literature, and papers in any language), and we had few exclusion criteria. We translated several papers into English for full-text review and we also examined the references of included reviews and book chapters to ensure we did not miss any relevant studies. In terms of guideline development, conflict of interest statements were reviewed by an impartial chair (MB) in order to address any biases. We had an initial virtual meeting to ensure that the research questions were unanimously agreed upon by the panel, as well as a second virtual meeting to discuss our recommendations, which was followed by an anonymous voting procedure. Furthermore, our panel included the voices of various stakeholder groups including researchers, clinicians, policymakers, parents, and those with lived experience of receiving treatment for an ED.

### Limitations

Although thorough, our search strategy had limitations. We were unable to retrieve seven citations as full text articles as they could not be located. We also attempted to examine sex differences, but the numbers of male subjects were so small that no conclusions could be drawn, and studies did not comment on the impact of sex on virtual care. While we searched the literature thoroughly for virtual day hospital and virtual meal support, we were unsuccessful in finding any articles on these topics. We found one mixed methods study related to virtual meal support training (pre-recorded videos) for caregivers. Furthermore, virtual care presents some difficulties related to diagnostic certainty. Many studies enrolled participants based on symptom evaluation using instruments rather than diagnostic interview, with study inclusion criteria also bridging DSM-IV and DSM-5 criteria. Therefore, recommendations for specific ED diagnoses may be seen as suggestive rather than definitive.

While research about moderated online caregiver forums and support groups indicated that they are important sources of caregiver support, especially with resources being scarce during the COVID-19 pandemic, there is insufficient evidence of their impact on patient outcomes, and therefore are not being recommended as alternatives to treatment. Similarly, although self-help was not the main focus of our recommendations, the panel agreed a priori to our literature search that self-help resources such as books and manuals (including unguided self-help) should be examined as possible sources of support. After review and discussion of the findings, the panel concluded that book and manual self-help resources should be considered as adjuncts to virtual care, rather than treatment. Despite these limitations, these guidelines represent a significant step forward in adopting virtual care in the field of EDs for treatment among treatment-seeking children, adolescents, emerging adults and their families.

### Future directions

Several gaps were noted by the guideline panel, which should be a focus for future study. These gaps include the impact of sex, gender, and underserved populations on virtual care among children, adolescents and emerging adults with EDs, as well as the efficacy of more intensive treatments such as virtual day hospital, or virtual meal support therapy. Mixed methods studies may be a useful study design for these understudied areas, to understand both quantitative and qualitative impacts. There were several other gaps identified by the panel as needing further study. The panel was unable to make any recommendations for adjunctive email therapy and/or online counselling, and text messaging therapy. While some encouraging evidence was found in these areas for children, adolescents and emerging adults with AN, BN, BED, and EDNOS, the panel concluded that these therapies are promising in terms of possible adjuncts to treatment, however, should not be recommended without further study. The panel suggested that creative interventions be developed for those on a waiting list or transitioning in levels of care. Such a possibility might be self-compassionate letter writing unguided self-help (adapted from compassion-focused therapy) for emerging adults, [80] but again more research is required to support this recommendation. Furthermore, there was some positive evidence for telehealth cognitive and behavioural treatment for children and adolescents with ARFID and OSFED, but additional research is also required to draw conclusions, as sample sizes in these studies were small. Finally, research on telehealth family interventions for ARFID and OSFED is lacking but should be a focus of further study.

## Conclusions

Our Canadian Practice Guidelines for virtual care during COVID-19 and beyond recommend several options for treatment (or adjuncts to treatment), as well as some general principles, and areas for future research. A strong recommendation was supported in favour of in-person medical evaluation, when necessary, for children, adolescents, and emerging adults with EDs; and that equity-seeking groups and marginalized youth should be provided equal access to treatment. In addition, for emerging adults, internet CBT-based guided self-help for AN, BN, BED, and EDNOS was strongly recommended. Additionally, for this age group, weak recommendations were generated for CBT-based group internet interventions as treatment adjuncts for high body dissatisfaction, internet-based relapse prevention MANTRA guided self-help for AN, telehealth relapse prevention using MANTRA for AN, and guided CBT-based smartphone apps as treatment adjuncts for AN. For children and adolescents with AN, weak recommendations were supported for telehealth FBT, and online guided parental self-help FBT. In terms of caregiver outcomes for caregivers of emerging adults, guided parental self-help CBT was strongly recommended, while unguided caregiver psychoeducation self-help was weakly recommended. For caregivers of children and adolescents with EDs, weak recommendations were supported for virtual parent meal support training, as well as moderated online caregiver forums and support groups. Future research is required to understand the impact of sex, gender, race, socioeconomic status, and other factors on virtual care among children, adolescents and emerging adults with EDs; as well as research on more intensive services such as virtual day hospitals.

## Data Availability

Not applicable.

## Appendix A

**Table:** Summary of included studies regarding the impact of COVID-19 on eating disorders

Reference	Type of Study	Age group	ED diagnosis	Sample size	Main findings
Barney et al. 2020 [47]	Descriptive	18–25 years	All EDs	Not specified	Telemedicine visits increased from 0 to 97% in 1 month. Providers noted ED patients particularly benefitted from telemedicine as they are already often referred to clinics from a much wider geographic range than typical primary care patients; also created flexibility for families. Challenges: privacy (so providers encouraged headphones), inability to assess anthropometric data (so providers trained families to take weights at home or partnered with local primary care providers to collect data).
Bryant-Waugh, 2020 [53]	Editorial	Any age	ARFID	Not applicable	When treating individuals with ARFID, especially during COVID-19, collaborative work is encouraged between clinicians, researchers, and those with lived experience, to better understand epidemiology, etiology, impact, and co-occurring conditions. It is important to foster communities of kindness towards ARFID (during and beyond COVID), which involves carefully listening to individuals, family members, and partners of those affected by ARFID.
Clark-Bryan et al. 2020 [49]	Qualitative	18–25 years	AN	*n* = 21 individuals with AN; *n* = 28 caregivers	People with AN: reduced access to ED services (reliance on remote support which was received with varying levels of acceptability); disruption to routine; reduced motivation for recovery; heightened psychological distress and ED symptoms; increased attempts at self-management in recovery. Caregivers: fears of premature discharge of patient; increased demands to manage patient needs; new challenges around managing patient well-being (spotting signs of relapse); new opportunities (gratitude for increased time at home).
Davis et al. 2020 [45]	Descriptive	< 18 years	All EDs	Not specified	Scaling up of outpatient treatment delivered via telehealth. Patients and families had heightened health-related fear. In the community, school counsellors and community social workers partnered with psychologists to support ED patients. Caregivers working greater hours due to the pandemic had difficulties with child meal supervision and being involved in care.
Emans et al. 2020 [48]	Commentary	18–25 years	All EDs	Not applicable	Some ED programs enlisted parents to monitor weight and vital signs. Some ED visits for patients were co-managed with local primary care providers until in-person visits could be arranged.
Janse van Rensburg, 2020 [52]	Letter to the editor	Any age	All EDs	Not applicable	ED behaviours may be the only coping tool available for those with EDs during COVID-19, so a harm-reduction, person-centered approach is proposed. Goal: decrease likelihood of mortality by maintaining ED behaviours but increasing their safety; allowing the individual to set boundaries about what behaviours they are not willing to give up at that time.
Matheson et al. 2020 [26]	Descriptive	< 18 years	AN, BN, ARFID	Not applicable	Challenges: difficulties in ensuring accurate communication when monitoring remotely, patients not physically present for weigh-ins, privacy, hard to build rapport virtually, lack of family involvement in FBT via video, difficult to conduct family meal session of FBT, families abruptly leaving virtual sessions.
Murphy et al. 2020 [51]	Descriptive	Any age	All EDs	Not applicable	CBT-E is well suited for remote delivery during COVID-19 if adapted appropriately: have an assessment phase with patient to discuss a crisis plan, use video-call platforms instead of audio calls, keep virtual sessions the same length as in-person sessions, explain to patients how online sessions will work
Waller et al. 2020 [50]	Descriptive	Any age	All EDs	*n* = 22 clinicians	Patients may view telehealth as ‘second best’, so clinicians need to stress that it is ‘business as usual’. Reliable programs (e.g. Zoom, Microsoft Teams) should be used over unreliable programs (e.g. FaceTime). COVID-19-specific ED psychoeducation should be included in CBT sessions.
Walsh and McNicholas, 2020 [46]	Descriptive	< 18 years	AN	Not applicable	Weekly FBT sessions were held by telephone or Zoom, caregivers were required to weigh their child at home using a home scale, record food intake, estimate energy expenditure, collect vital signs; caregiver burden increased.
Wood et al. 2020 [44]	Case report in a descriptive study	< 18 years	AN	*n* = 1	Using telehealth FBT, the case achieved prescribed calorie goals, reduced/eliminated purging, reduced emotional outbursts, engaged in care, and avoided re-hospitalization and other higher levels of care.
Weissman et al. 2020 [18]	Editorial	Any age	All EDs	Not applicable	Most ED patients have favourable attitudes towards e-therapy and willingness to engage in it, but few express a preference for it over in-person therapy. Clinicians are concerned that patients may expect them to handle their issues during off-work hours, contributing to a higher workload.
Weissman et al. 2020 [55]	Cross-sectional	Any age	All EDs	*n* = 187 researchers	During COVID-19, many ED researchers moved studies online and/or had to shut down part of their research. Researchers reported a need to advocate for ED research related to COVID-19. Strategies for dealing with research disruptions: employing technology, reprioritizing goals/tasks (e.g. changing future research directions), encouraging collaboration.
Weissman et al. 2020 [54]	Editorial	Any age	All EDs	*n* = 187 researchers	Researchers associated with the International Journal of Eating Disorders requested: more flexible timeframes to submit or review revisions, fast-tracking of COVID-19-related studies for EDs, research conducted on the COVID-19 impact on ED research, standards for publication to be maintained.

## Appendix B

**Table:** Summary of included studies regarding virtual care and eating disorders

Reference	Type of Study	Age group	ED diagnosis	Sample size	Intervention	Main findings
Aardoom et al. 2014 [86]	Cross-sectional	18–25 years	Not specified; those experiencing ED symptoms	*n* = 311	Moderated online forums	Exchanging information, finding recognition, and sharing experiences were the empowering processes often reported; most pronounced empowering outcome was feeling better informed. To a smaller degree, increased help-seeking behavior, optimism and control, and confidence in treatment and relationship with the therapist.
Aardoom et al. 2016 [61]	RCT	18–25 years	AN, BN, BED	*n* = 87 Featback; *n* = 88 Featback +low-intensity therapist support; *n* = 89 Featback+ high-intensity therapist support; *n* = 90 waitlist control	Internet CBT-based guided self-help (Featback)	3 Featback conditions were superior to control in reducing ED psychopathology, and ED symptoms (e.g. cessation from binge eating and purging). No added value of therapist support.
Aardoom et al. 2016 [64]	RCT (economic evaluation)	18–25 years	AN, BN, BED	*n* = 87 Featback; *n* = 88 Featback +low-intensity therapist support; *n* = 89 Featback+ high-intensity therapist support; *n* = 90 waitlist control	Internet CBT-based guided self-help (Featback)	No significant differences between the study conditions were found regarding quality-adjusted life-years and societal costs, but the mean costs per participant were lowest in the Featback condition with low-intensity therapist support. Featback seems to be cost-effective compared to waitlist.
Aardoom et al. 2017 [65]	RCT	18–25 years	AN, BN, purging disorder, EDNOS	*n* = 60 Featback; *n* = 70 Featback +low-intensity therapist support; *n* = 71 Featback + high-intensity therapist support; *n* = 72 waitlist control	Internet CBT-based guided self-help (Featback)	Baseline levels of ED psychopathology were found to moderate intervention response. Featback with and without therapist support is particularly useful in improving bulimic psychopathology but may be less suitable in improving severe anorectic psychopathology.
Anastasiadou et al. 2019 [87]	Qualitative	< 18 years	AN, BN, EDNOS	*n* = 9 patients; *n* = 11 mHealth experts; *n* = 10 healthcare professionals; *n* = 8 ED specialists	Smartphone applications (TCApp)	Professionals consider mHealth as difficult to obtain and use (barriers = lack of time). According to patients and ED specialists, app was easy to use and useful. Patient barriers were lack of personalization and lack of motivational and interactive components. Patients disliked web-based chat option with ED specialist.
Anderson et al. 2017 [56]	Open trial (feasibility)	< 18 years	AN, atypical AN	*n* = 10	Telehealth FBT	Significant weight gain was achieved from baseline to end of treatment and baseline to 6-month follow-up.
Bailey et al. 2002 [96]	Case report	< 18 years	AN	*n* = 1	Email and/or online counselling	Patient emailed psychiatrist several times/week, describing food eaten, mood, psychological changes, etc. Patient generally described email as a positive treatment adjunct that was good for self-reflection, but had issues with privacy, having “one more thing to do”.
Barney et al. 2020 [47]	Descriptive	18–25 years	All EDs	Not specified	In-person medical evaluation	If a patient was clearly engaging in concerning behaviours (e.g increased restricting, purging, over exercising) or the provider or caregiver had a high index of suspicion for medical deterioration, the patient was asked to come in for limited in-person sessions, rather than continuing with virtual care visits.
Bauer et al. 2003 [94]	Case report	18–25 years	BN	*n* = 2	Text messaging	Generally positive outcomes of using the aftercare intervention (e.g. no bingeing/purging reported) over the course of 12 to 14 weeks of use. Some issues still present (e.g. low body satisfaction).
Billeci et al. 2015 [118]	Open trial (pilot)	< 18 years	AN	*n* = 27 with AN; *n* = 15 healthy controls	Home monitoring	Wearable sensors combined with wireless technologies were feasible in young adolescents with AN, providing useful information on heart rate and heart rate variability. Instruments proved to be suitable in AN subjects; their applications could open new approaches of vital signs monitoring at home, but by a clinician rather than a caregiver.
Binford Hopf et al. 2013 [116]	Open trial (pilot)	< 18 years	AN	*n* = 13	Moderated online caregiver forums	High chat programme satisfaction ratings (91.7%). Parents reported looking forward to chat sessions and would recommend to others. Vast majority reported that the chat helped them cope with child’s ED and to implement FBT; overall rated as accessible, convenient, and easy to use
Bloomfield et al. 2019 [59]	Case report	< 18 years	ARFID	*n* = 1	Telehealth CBT	Increased frequency of bites of nonpreferred foods consumed.
Cairns et al. 2007 [109]	Mixed methods	< 18 years	AN, BN, EDNOS	*n* = 40	Unguided caregiver self-help using web-based and/or pre-recorded videos focused on psychoeducation and communication skills	Program was reported as informative, convenient and was well-received by families. Many parents reported that the resources helped them be more understanding and patient with their youth and the recovery process.
Carrard et al. 2011 [69]	Open trial	18–25 years	BN, subthreshold BN	*n* = 127	Internet CBT-based guided self-help	Severity of ED symptoms (e.g. bingeing) and general psychopathology improved significantly
Cavazos-Rehg et al. 2020 [88]	Mixed methods	< 18 years	AN, BN, BED, subclinical BN or BED, purging disorder, unspecified feeding or eating disorder	*n* = 366	Smartphone applications	Those with more severe manifestations of illness were more interested in trying the app. Primary reasons for unwillingness: wanting to retain privacy, felt parents lack awareness of mental health issues, concerns of parents’ reaction, poor parent-teen relationship, feeling of parent not wanting child to participate.
Dimitropoulos et al. 2019 [110]	RCT (feasibility)	18–25 years	AN, BN	*n* = 23 caregivers in web-based intervention; *n* = 27 caregivers in workshop intervention	Unguided caregiver self-help using web-based and/or pre-recorded videos focused on psychoeducation and communication skills	From baseline to end of intervention, small between-group effect sizes were observed for changes in caregiver accommodation, problem-solving abilities, the quality of psychological health, and quality of social relationships, favouring web intervention, and changes in expressed emotion in family context, caregiver burden, perceived stress, and the quality of the environment, supporting the workshop intervention.
Duncan et al. 2014 [60]	Case report	< 18 years	EDNOS	*n* = 1	Telehealth CBT	Increase in food intake, improvements in growth, and reduced depression scores post-treatment.
Dunn et al. 2006 [82]	RCT	18–25 years	BN, BED	*n* = 45 self-help only; *n* = 45 MET adjunct + self-help	MET and self-help book (Overcoming Binge Eating)	MET intervention resulted in increased readiness to change for binge eating compared with the self-help only intervention. Few differences between conditions for eating attitudes and frequency of binge eating and compensatory behaviours (both groups had improvements). Within-group effects indicated that those in MET condition had significant reductions in binge eating, compensatory behaviours, and maladaptive attitudes; changes in self-help only were not significant.
Emans et al. 2020 [48]	Commentary	18–25 years	All EDs	Not specified	In-person medical evaluation	During COVID-19, ED programs utilized weight and heart rate criteria to define urgency and need for in-person medical assessments. Some programs established ‘vital signs clinics’, where patients come in for a heart rate, blood pressure, and weight assessment, then either followed or preceded by a telehealth visit.
Fernandez-Aranda et al. 2009 [70]	Observational (controlled study)	18–25 years	BN	*n* = 31 intervention; *n* = 31 waitlist control	Internet CBT-based guided self-help	Significant decreases at follow-up in psychopathological levels, binge eating, and vomiting, favouring intervention group over controls.
Fichter et al. 2008 [78]	Observational (controlled study)	18–25 years	AN	*n* = 51 manual intervention; *n* = 51 waitlist control	Manual CBT-based guided self-help	Duration of inpatient treatment was significantly shorter (by 5.2 days) among participants that received guided self-help. Body image, slimness ideal, general psychopathology and some bulimic symptoms improved significantly for intervention group. BMI did not differ significantly between groups, but intervention group showed more weight gain.
Fichter et al. 2011 [66]	RCT (feasibility)	18–25 years	AN	*n* = 128 VIA intervention; *n* = 130 treatment as usual	Internet CBT-based guided self-help (VIA)	VIA program was highly feasible with moderate overall dropout rate (15.5%); program was well-received.
Fichter et al. 2012 [63]	RCT	18–25 years	AN	*n* = 128 VIA intervention; *n* = 130 treatment as usual control	Internet CBT-based guided self-help (VIA- Virtual Intervention for AN)	VIA intervention group gained significantly more weight than controls. Group by time comparisons for eating-related cognitions, behaviours, and general psychopathology showed a significantly more favourable course in the intervention for bulimic symptoms.
Fichter et al. 2013 [67]	RCT (follow-up study)	18–25 years	AN	*n* = 92 VIA intervention; *n* = 120 control	Internet CBT-based guided self-help (VIA)	Most variables showed improvements in VIA intervention, but only some reached statistical significance (e.g. bulimic behaviour). Very good results for BMI for full completers (9 months).
Fitzsimmons-Craft et al. 2019 [90]	Open trial	18–25 years	Clinical or subclinical ED, excluding AN	*n* = 13 universities	Smartphone applications (Student Bodies-ED)	SB-ED results were encouraging as restrictive eating and binge eating significantly decreased over the course of users’ time in the intervention but vomiting and diet pill/laxative use were not found to significantly decrease (but reports of these behaviours were very low).
Giel et al. 2015 [58]	Open trial (pilot)	18–25 years	AN	*n* = 12	Relapse prevention (MANTRA) via telehealth	At post-treatment, participants’ BMI increased, and eating, shape, and weight concerns (EDE-Q scores) decreased, compared to baseline.
Goldfield and Boachie, 2003 [57]	Case report	< 18 years	AN	*n* = 1	Telehealth FBT	Significant weight gain was achieved from baseline to end of treatment
Gollings et al. 2006 [83]	RCT (pilot)	18–25 years	Probable BN and/or high body dissatisfaction	*n* = 19 face-to-face delivery; *n* = 21 internet-delivery	CBT-based group internet intervention (‘Set your body free’)	Large improvements in body dissatisfaction (BSQ, BIAQ scores) and dietary restraint (DEBQ-R scores) in face-to-face and internet-delivery groups. No significant differences between delivery modes at post-treatment or 2-month follow-up.
Grover et al. 2011 [113]	RCT (pilot)	18–25 years	AN	*n* = 33 carers in web-based intervention; *n* = 30 carers in hotline control	Guided parental self-help CBT (skills training approach + workbook or CBT-based online modules	Compared with hotline control, the web-based intervention had a significantly greater positive impact on caregivers’ depression and anxiety (primary outcome), with similar trend in caregivers’ expressed emotion. Other secondary outcome measures were also reduced across both groups, but there was no significant difference between groups (did not favour web-based intervention).
Grunwald and Wesemann, 2006 [103]	Cross-sectional	18–25 years	BN, BED, AN	*n* = 240 datasets of affected persons; *n* = 85 datasets of relatives	Email and/or online counselling	22.5% of affected persons and 49.4% of relatives stated that answers provided by online consultants led to a better understanding EDs. 55.4% of affected persons and 81.2% of relatives had not turned to professional help before the use of the online service. 20% of affected persons went to see a therapist as a result of online consultation.
Grunwald and Wesemann, 2007 [104]	Cross-sectional	18–25 years	AN, BN	*n* = 2176 emails	Email and/or online counselling	Consulting service was predominantly used by persons suffering from BN or their friends/families. Content of emails also included seeking info about EDs, asking for help to find specialized clinics/therapists.
Hoyle et al. 2013 [114]	RCT (feasibility)	18–25 years	AN	*n* = 19 OAO+ guidance intervention; *n* = 18 OAO only intervention	Guided parental self-help CBT (skills training approach + workbook or CBT-based online modules	Significant reductions were found for caregiver intrusiveness, negative experiences of caregiving, and the impact of starvation and guilt. Within group effect sizes: mixed feelings with respect to whether greater benefits were conferred for caregivers receiving clinician guidance. Individuals with AN did not perceive that their caregiver’s levels of expressed emotion had significantly changed but decreases in perceived intrusiveness of the caregiver by the person with AN had a large effect size. Caregivers’ ED symptom impact scale scores were also reduced but did not differ between conditions.
Jones et al. 2012 [108]	Open trial (pilot)	< 18 years	AN, or high-risk for AN	*n* = 12 diagnosed with AN, *n* = 12 at risk for AN, *n* = 22 at high-risk for AN	Online guided parental self-help – FBT	Children remained stable or increased in ideal body weight and reported decreased ED attitudes and behaviours. Parents with at-risk children rated the programme more favourable than parents with children who had diagnosed AN.
Kelly and Waring, 2018 [80]	RCT (feasibility)	18–25 years	AN, atypical AN	*n* = 20 intervention; *n* = 20 waitlist control	Internet-delivered self-compassionate letter writing unguided self-help	Intervention appeared to be acceptable, feasible, and efficacious; greater increases in self-compassion and greater decreases in shame and fears of self-compassion comparted to waitlist controls. Changes in EDE-Q scores and readiness to get help for one’s weight did not differ between conditions.
Kendal et al. 2017 [85]	Qualitative	< 18 years	All EDs	*n* = 119	Moderated online forums	How young people use the forum: taking on the role of a mentor, online discussion forum as a safe space, friendship within the online forum, flexible help, peer support for recovery and relapse prevention. Moderated forums may foster a supportive environment.
Kindermann et al. 2016 [72]	Case report	18–25 years	BN symptoms	*n* = 1	Internet CBT-based guided self-help (ProYouth)	Symptoms did not improve following intervention, but case reported the program contributed to decision to pursue face-to-face treatment.
Kundiger et al. 2010 [105]	Mixed methods	18–25 years	AN, BN	*n* = 803	Email and/or online counselling	Main group of users were females, persons affected, relatives/friends of those affected. 24.5% of inquiries asked for contact info for local mental health professionals or institutions. For many, this was first point of contact with mental health professional.
LaMarre et al. 2015 [115]	Qualitative	< 18 years	Not specified	n = 5	Moderated online caregiver forums	Main themes: (1) the importance of support; (2) shifts in parenting. Blogs detailed how parents actively seek to meet needs during a difficult time using online interactions to bolster sources of support that exist offline. Blogs were used as adjunct support for families.
Lang et al. 2015 [81]	Qualitative	18–25 years	AN, EDNOS	*n* = 6	Manual-based CRT	High level of satisfaction and acceptability for self-help format and working with parents; parents and individuals stated they would recommend it, but suggested computerized CRT for the future.
Lock et al. 2017 [107]	Case series	< 18 years	AN	*n* = 19	Online guided parental self-help – FBT	Patients experienced weight gain similar to clinician-delivered FBT programs and ED-related psychopathology improved by end of treatment.
Marx and Cohen, 2006 [117]	Case report	12–22 years	All EDs	*n* = 6	Moderated online caregiver forums	Teleconferencing was seen as an effective modality to help parents who feel emotionally isolated. While in-person meetings may be more ideal, teleconferencing may be the only way for some parents to have a voice in helping their child, and themselves.
Matheson et al. 2020 [26]	Descriptive	< 18 years	AN, BN, ARFID	Not applicable	Home monitoring	Caregivers were taught how to weigh child at home with the therapist virtually present for telehealth FBT.
Moessner and Bauer, 2012 [101]	Qualitative	18–25 years	AN, BN	*n* = 238	Email and/or online counselling	Rated very positively; majority would use again and recommend to others. Main reasons for using online services: anonymity, fast and easy contact, competence related to ED, and free
Murdoch and Connor-Greene, 2000 [99]	Case report	18–25 years	Likely BN or AN-purging type	*n* = 1	Email and/or online counselling	Email as a therapy adjunct served as an important outlet and means of coping; created a greater sense of trust with therapist.
Neumayr et al. 2019 [89]	RCT (pilot)	18–25 years	AN	*n* = 20 Recovery Record+ treatment as usual *n* = 20 treatment as usual	Smartphone applications (Recovery Record)	Non-significant small to moderate between-group effect sizes favouring Recovery Record group over treatment as usual only group (in BMI and EDE-Q scores). 6-month follow-up: effects wore off and no significant differences between the intervention and control groups were evident.
Nevonen et al. 2006 [71]	Case series	18–25 years	BN, EDNOS	*n* = 38	Internet CBT-based guided self-help	Significant decrease in core ED symptoms (vomiting, dietary restraint, weight phobia) except for binge eating and excessive exercise. When bingeing and vomiting decreased, exercise increased ➔ participants were changing their ways of compensating.
Nitsch et al. 2016 [92]	Mixed methods (feasibility)	18–25 years	Those with body image concerns, disordered eating symptoms, but excludes full syndrome AN	*n* = 9	Smartphone applications (Student Bodies-ED)	Participants were satisfied with the overall usability of the program (average usability score: 77.5/100 and improved to 83.1/100 after modifications).
Paxton et al. 2007 [84]	RCT	18–25 years	Probable BN and/or high body dissatisfaction	*n* = 42 face-to-face delivery; *n* = 37 internet-delivery; *n* = 37 delayed treatment control	CBT-based group internet intervention (‘Set your body free’)	Large improvements in body dissatisfaction (BSQ, BIAQ scores) and dietary restraint (DEBQ-R scores) in face-to-face and internet-delivery groups compared with control. Post-treatment improvements were greater in face-to-face group than internet group, but gains made in both groups were maintained at 6-month follow-up, and groups were not clearly different from each other at 6-month follow-up.
Prescott et al. 2019 [97]	Qualitative	< 18 years	All EDs	*n* = 4	Email and/or online counselling	Users suggested the forums were supportive environments where they felt able to interact with others, share advice, ask questions- making them feel less alone and more connected to others.
Pretorius et al. 2009 [68]	Open trial (feasibility)	18–25 years	BN, EDNOS with bulimic features	*n* = 101	Internet CBT-based guided self-help (Overcoming Bulimia Online)	Significant improvements in ED symptoms (decreased objective binge eating and vomiting) from baseline to 3 months and maintained at 6 months.
Pretorious et al. 2010 [73]	Qualitative	18–25 years	BN, atypical BN, EDNOS-BN	*n* = 11	Internet CBT-based guided self-help (Overcoming Bulimia Online)	Participants liked the programme for its accessibility, flexibility, support, and information. Some used the intervention as a stepping-stone to further treatment.
Quadflieg et al. 2017 [111]	RCT	18–25 years	AN, BN, atypical AN, atypical BN, EDNOS	*n* = 147 intervention; *n* = 138 control	Unguided caregiver self-help using web-based and/or pre-recorded videos focused on psychoeducation and communication skills	Acceptability of the intervention was high. Caregivers’ burden and psychological distress were reduced by the intervention, but not caregivers’ accommodating behaviours.
Robinson and Serafty, 2001 [106]	Open trial (pilot)	18–25 years	BN, BED, EDNOS	*n* = 23	Email and/or online counselling	At 3-month follow-up, there were significant improvements in depressive and bulimic symptoms. Email therapy was seen as acceptable to patients who may be reluctant to seek treatment, and may provide a useful treatment for BN.
Sanchez-Ortiz et al. 2011 [62]	RCT	18–25 years	BN, EDNOS	*n* = 38 iCBT; *n* = 38 waitlist/delayed treatment control	Internet CBT-based guided self-help (Overcoming Bulimia Online)	Significantly greater reductions in EDE scores, binge eating episodes, all other ED variables in intervention group immediate iCBT access. Gains maintained or continued to improve at follow-up.
Sanchez-Ortiz et al. 2011 [74]	Qualitative	18–25 years	BN, EDNOS	*n* = 9	Internet CBT-based guided self-help (Overcoming Bulimia Online)	Program received positively as a way of fitting treatment into busy lives. Email support was perceived as an essential part of treatment and a source of motivation
Sanchez-Ortiz et al. 2011 [102]	Qualitative	18–25 years	BN, EDNOS	*n* = 71	Email and/or online counselling	105 (14.7%) of the emails that therapists sent contained at least 1 CBT comment, 679 (95.4%) contained at least 1 supportive comment, and 97 (13.6%) contained at least 1 technical/study-related comment.
Sansone, 2001 [100]	Case report	18–25 years	AN	*n* = 4	Email/and or online counselling (without clinician)	Participants described positive feelings about program and gained support. All participants progressed well in treatments. Risks may include email partners comparing weights amongst each other.
Sepulveda et al. 2008 [112]	Mixed methods (pilot)	< 18 years	AN, BN	*n* = 16	Guided caregiver self-help – Skills	Caregivers expressed high levels of satisfaction with most aspects of DVD and coaching training. Caregiver general distress (General Health Questionnaire) decreased significantly from baseline to post-intervention. Caregiver psychological distress and depression after intervention did improve but not statistically significant.
Schmidt et al. 2017 [79]	RCT	18–25 years	AN	*n* = 86 patients and *n* = 134 caregivers in ECHO intervention +treatment as usual; *n* = 92 patients and *n* = 134 caregivers in treatment as usual	Guided parental self-help CBT (skills training approach + workbook or CBT-based online modules	Caregivers in the ECHO group spent less time caregiving and had less burden and expressed emotion at discharge and/or 6-month follow-up; greater reduction in ED symptom impact scale scores in the intervention group compared to control. At 6 months, patients experienced a decrease in ED symptoms and an improved quality of life.
Schmidt et al. 2017 [79]	RCT (feasibility)	18–25 years	AN	*n* = 24 iMANTRA +treatment as usual; *n* = 17 treatment as usual	Internet-based MANTRA guided self-help	6 months: little difference between iMANTRA group and treatment as usual group. 12 months: iMANTRA group had higher BMI and lower scores on depression, anxiety, and stress scales; confidence intervals were wide and overlapped with 0.
Shingleton et al. 2016 [93]	Open trial (feasibility)	18–25 years	AN, subclinical AN, BN	*n* = 12	Text messaging	Well accepted and feasible. Text messages did not impact behavioural outcomes (dietary restraint and calorie intake); had mixed effects on motivation to change dietary restraint. In response to text messages, underweight participants had an increased desire to restrict and increased action toward reducing restriction, whereas normal weight participants reported only increased action toward restricting.
Tregarthen et al. 2015 [91]	Case report	18–25 years	All EDs	*n* = 108,996	Smartphone applications (Recovery Record)	In 2 years, 108,996 downloaded the app; 89% monitored at least 3 meals per day; 67% continued using app 30 days later. Of 2503 ratings, 84% rated it 5/5. Since ~ 50% users reported that they do not currently receive treatment, app may be effective in reaching an underserved population, but raises concern that for some, it may replace seeking treatment, which might be clinically warranted.
Wagner et al. 2013 [75]	RCT	18–25 years	BN, EDNOS	*n* = 70 INT-GSH, *n* = 56 BIB-GSH	CBT-based bibliotherapy	Significant improvements in ED symptoms in both groups. No significant difference regarding outcome between delivery modes. Email guided self-help (delivered via internet or bibliotherapy) is equally effective for adolescents and adults.
Wagner et al. 2013 [76]	RCT (follow-up study)	18–25 years	BN, EDNOS	*n* = 70 INT-GSH, *n* = 56 BIB-GSH	CBT-based bibliotherapy	Greatest improvements after 4 months. Continued reduction in ED symptomatology at month 7 and 18. No differences regarding outcome between the 2 groups were found.
Wagner et al. 2015 [77]	RCT (follow-up study)	18–25 years	BN, EDNOS	*n* = 70 INT-GSH, *n* = 56 BIB-GSH	CBT-based bibliotherapy	Higher motivation, lower frequency of binge eating, and lower body dissatisfaction at baseline predicted good outcomes after the end of both treatments.
Walsh and McNicholas, 2020 [46]	Descriptive	< 18 years	AN	Not applicable	Home monitoring	For telehealth FBT, caregivers are required to collect weekly weights, record food intake, estimate energy expenditure, and collect vital signs of the child.
Wood et al. 2020 [44]	Descriptive study	< 18 years	AN	Not applicable	Home monitoring	For telehealth FBT: the patient’s heart rate, blood pressure, and weight were monitored by a parent at home. The clinician visually inspected the patient via videoconferencing and had frequent follow-up visits to allow for regular assessment of progress.
Yager, 2001 [98]	Case report	18–25 years	AN	*n* = 3	Email and/or online counselling	Email seemed to have helped patients – no negative effects experienced by patients or clinician. Email had excellent patient acceptability and adherence.
Yager, 2003 [95]	Case report	< 18 years	AN	*n* = 2	Email and/or online counselling	Weight gain achieved in 1/2 patients. Potential benefits of email adjunct: flexibility, increased contact with physician, patients can become more aware of their ED behaviours by documenting them.

## Abbreviations

AGREE II: Appraisal of guidelines for research and evaluation
AN: Anorexia Nervosa
ARFID: Avoidant/Restrictive Food Intake Disorder
BED: Binge Eating Disorder
BIAQ: Body Image Avoidance Questionnaire
BIB-GSH: Bibliotherapy guided self-help
BMI: Body mass index
BN: Bulimia Nervosa
BSQ: Body Shape Questionnaire
BULIT-R: Bulimia Test-Revised
CBT: Cognitive behavioural therapy
CBT-E: Enhanced cognitive behavioural therapy
CRT: Cognitive remediation therapy
DASS-21: Depression, Anxiety, and Stress Scales
DEBQ-R: Dutch Eating Behaviour Questionnaire Restraint Scale
ECHO: Experienced Carers Helping Others
ED: Eating disorder
EDE-Q: Eating Disorder Examination-Questionnaire
EDI: Eating Disorders Inventory
EDNOS: Eating Disorder Not Otherwise Specified
FBT: Family-based treatment
FEAST: Families Empowered and Supporting Treatment of Eating Disorders
GDP: Guideline development panel
GRADE: Grading of Recommendations, Assessment, Development, and Evaluation system
GUIDE-M: Guideline implementability for decision excellence model
INT-GSH: Internet guided self-help
iMANTRA: internet-based Maudsley Model of Anorexia Nervosa Treatment for Adults
MANTRA: Maudsley Model of Anorexia Nervosa Treatment for Adults
MET: Motivational Enhancement Treatment
OAO: Overcoming Anorexia Online
OSFED: Other Specified Feeding and Eating Disorder
PRISMA: Preferred reporting items for systematic reviews and meta-analyses
RCT: Randomized controlled trial
SD: Standard deviation
SEED: Short Evaluation of Eating Disorders
VIA: Virtual Intervention for Anorexia Nervosa
WHO: World Health Organization
